# Super enhancer lncRNA RP11-54O7.17 regulates the proliferation and metastasis of triple-negative breast cancer by targeting lysosomal degradation of S100A4

**DOI:** 10.1038/s41419-025-08072-3

**Published:** 2025-10-31

**Authors:** Hongtao Hu, Haoyang Bai, Chen Wang, Luyi Xi, Shasha Tian, Maowei Ni, Jiahui Lu, Hang Gao, Huajun Zhao

**Affiliations:** 1 School of Pharmaceutical Sciences, Zhejiang Chinese Medical University, Hangzhou, China; 2 Zhejiang Cancer Hospital, Hangzhou, Zhejiang China; 3 Academy of Chinese Medical Sciences, Zhejiang Chinese Medical University, Hangzhou, Zhejiang China

**Keywords:** Breast cancer, Cell growth, Drug screening, Chaperone-mediated autophagy

## Abstract

Triple-negative breast cancer (TNBC) is characterized by its high aggressiveness and treatment resistance, with limited therapeutic options and especially a lack of effective targeted therapeutic strategies. This study focuses on the role and regulatory mechanisms of super enhancer long non-coding RNA (SE-lncRNA) in TNBC. Through in-depth analysis of TCGA database, we revealed the specific expression pattern of SE-lncRNA in TNBC, and found that downregulation of RP11-54O7.17 was significantly correlated with poor prognosis of TNBC patients, which was experimentally verified. Both in vitro and in vivo results confirmed that RP11-54O7.17 overexpression effectively suppressed the proliferation and metastasis of TNBC. Further exploration showed that RP11-54O7.17 directly interacted with the S100A4 protein through its conserved L2b-type repeat structural fragment, promoted S100A4 binding to HSP70, targeting S100A4 degradation via the autophagy-lysosome pathway, which in turn blocked the activation of S100A4-STAT3 signaling axis. Moreover, RP11-54O7.17 delivered via liposome demonstrated significant anti-TNBC efficacy in an in vivo model without observing significant systemic toxicity. This study elucidates the regulatory role and molecular mechanism of RP11-54O7.17 in TNBC, which provides a strong scientific basis and potential therapeutic targets for the development of novel SE-lncRNA-based therapeutic strategies.

## Introduction

Breast cancer (BRCA) is one of the most common cancers in women in the last decades [1]. TNBC, which lacks the three markers of progesterone receptor (PR), estrogen receptor (ER), and human epidermal growth factor receptor 2 (HER2), is considered as the most aggressive subtype, accounting for about 16% of all BRCA cases [2]. TNBC is associated with a worse prognosis, a lower sensitivity to typical therapies, a higher rate of metastasis, and a greater tendency to recur compared to other subtypes of BRCA [3, 4]. Drugs targeting hormone receptors and HER2 are ineffective due to the lack of receptors, and the fact that less than 20% of TNBC patients are PD-L1-positive, hence targeted therapies and immunotherapies for TNBC are suboptimal [5]. The complex pathogenesis of TNBC results in more than 70% of patients facing challenges in obtaining favorable outcomes [6]. Therefore, it is of great importance to find effective TNBC targets and develop targeted therapies.

SE-lncRNA is lncRNA produced by the binding of SEs to transcription factors, with transcription activity highly dependent on SEs [7]. Contiguous aligned regions of enhancers spanning several thousand bases are known as SEs which can drive higher levels of transcription of target genes [8]. Our team had reported that inhibition of SEs-associated protein RARα phosphorylation and its kinase CDK7 existed anti-TNBC effects [9]. It was commonly assumed in the past that SE-lncRNAs were merely by-products of SEs activation without biological functions, but further research has shown that lncRNAs are more preferentially located near SEs and interacted with cis-regulatory regions, enabling them to regulate the expression of neighboring genes due to their high spatial and temporal specificity [10]. SE-lncRNAs have been reported to play a key role in the malignant progression of cancer [11–13], involving cancer-related biological processes including cell proliferation, apoptosis, autophagy, epithelial mesenchymal transition, extracellular matrix remodeling and angiogenesis, etc [14, 15]. Currently, SE-lncRNAs are found to affect only the STAT signaling pathway which can serve as a biomarker for early BRCA [16], but SE-lncRNAs directly related to TNBC and their biological functions have not been reported, which makes it possible to explore the key mechanisms of SE-lncRNAs in TNBC, and develop a novel direction for the diagnosis and treatment of TNBC.

S100A4 is an EF-chiral calcium-binding protein that interacts with a variety of macromolecules, including proteins and nucleic acids, and participates in complex biological processes such as proliferation, metastasis, differentiation, inflammation and Ca^2+^ homeostasis [17]. S100A4 has been found to be commonly dysregulated in a variety of cancers including breast [18–20], liver [21], and ovarian [22] cancers. S100A4 is primarily highly expressed in stromal cells of the tumor microenvironment, where it works by promoting angiogenesis and recruiting immune cells to modify the tumor microenvironment and induce the release of some of the cytokines and growth factors that promote cancer development [23, 24]. Studies have shown that S100A4 is known to modulate the STAT3 signaling pathway for BRCA development [25] and that upregulation of S100A4 is a predictor of early BRCA metastasis and poor survival [18, 19]. Meanwhile, S100A4 has higher expression in TNBC compared with other subtypes of BRCA, and it promotes the malignant progression of TNBC through stimulatory effects [26]. Inhibition of S100A4 could reduce the ability of TNBC cells to metastasize and proliferate, and enhance the effects of chemotherapeutic agents such as doxorubicin [27, 28]. Therefore, targeted modulation of S100A4 is an effective strategy for the potential treatment of TNBC.

In the present study, we found that the SE-lncRNA expression profile of TNBC was significantly different from that of non-TNBC. The SE-lncRNA RP11-54O7.17, which was downregulated in TNBC and significantly correlated with the prognosis of TNBC, existed as a structural fragment with L2b-type repeats, and its overexpression was able to suppress the proliferation and metastasis of TNBC cells both in vivo and in vitro. Notably, the interaction between RP11-54O7.17 and S100A4 promoted the binding of HSP70 to S100A4, and destabilized the protein structure of S100A4 via the autophagy-lysosome pathway, which in turn suppressed the transcription of pro-carcinogenic downstream genes of the S100A4/STAT3 signaling pathway. In addition, we found that RP11-54O7.17 liposomes could also play a positive anti-TNBC role in vivo.

## Results

### SE-lncRNAs expression differentiates BRCA subtypes and predicts prognosis of TNBC patients

**Fig. 1:**
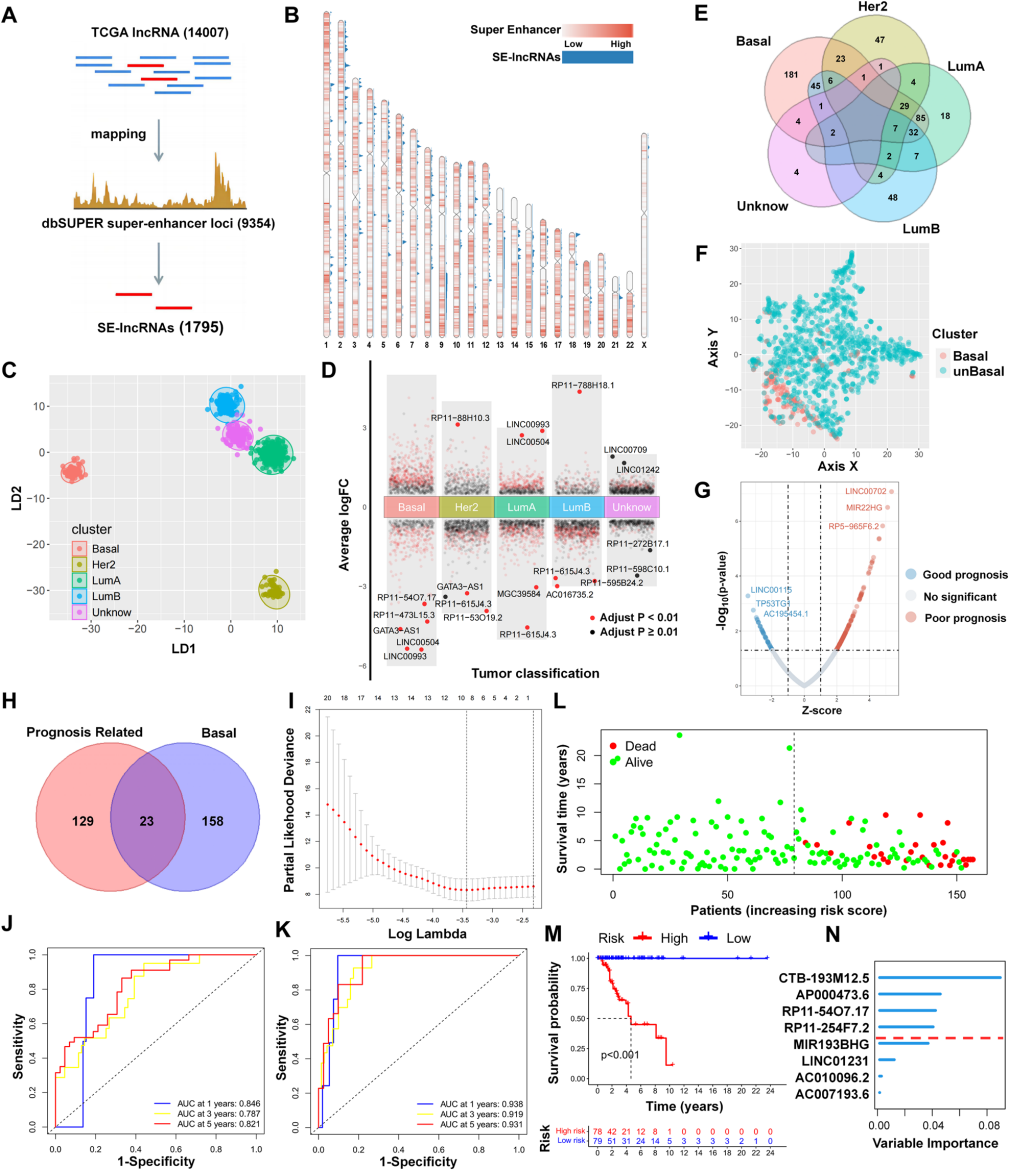
SE-lncRNAs expression differentiates BRCA subtypes and predicts prognosis of TNBC patients. **A** SE-lncRNAs identification process. **B** Chromosomal distribution of SE and SE-lncRNAs, the red extent represents the density of SEs and the blue peak height represents the density of SE-lncRNAs. **C** LDA cluster analysis of SE-lncRNA expression spectrum in BRCA patients. **D** Differential analysis of SE-lncRNA expression in various subtypes of BRCA. **E** Venn diagram of differentially expressed SE-lncRNAs. **F** t-SNE down-conversion analysis of SE-lncRNA expression spectrum. **G** SE-lncRNA volcano map related to BRCA prognosis. **H** Veen diagram of characteristic SE-lncRNAs correlated with TNBC prognosis. **I** LASSO sectional drawing of 23 SE-lncRNAs according to log(Lambda). **J** Multifactorial COX regression model of 1-, 3-, and 5-year survival prediction ROC curves. **K** Random forest model of 1-, 3-, and 5-year survival prediction ROC curves. **L** Scattergram of risk scores and patient survival. **M** Survival analysis based on risk scores. **N** Gene Importance in random forest model.

To investigate the role of SE-lncRNAs in TNBC, it is required to screen a reliable set of SE-lncRNA genes. After integrating the CHIP data of H3K27ac in the dbSUPER database (http://bioinfo.au.tsinghua.edu.cn/dbsuper/), 9354 super-enhancer regions were obtained, which were matched with the identified lncRNAs in the TCGA database (https://www.cancer.gov/ccg/research /genome-sequencing/tcga), and 1795 SE-lncRNAs were identified (Fig. 1A). Visualization of the density and location of SE and SE-lncRNAs by chromosome map (Fig. 1B) revealed that super-enhancer regions were present on all chromosomes, with the most SE-lncRNA expression present on chromosome 1.

Samples were subtyped using PAM50 based on the BRCA bulk transcriptomic data in TCGA database, where “Basal” was considered as the major TNBC subtype, and “Unknown” represented samples that could not be identified [29]. Cluster analysis of the SE-lncRNA expression spectrum data of BRCA patients in the TCGA database with the supervised LDA algorithm (https://www.stats.ox.ac.uk/pub/MASS4/) revealed that there were significant differences between different subtypes of BRCA (Fig. 1C), indicating that it is possible to distinguish between different subtypes of BRCA patients by SE-lncRNA expression spectrum. SE-lncRNAs differentially expressed in each subtype were screened by Limma package (http://bioconductor.org/packages/release/bioc/html/limma.html) and counted (Fig. 1D), showing that more differentially expressed SE-lncRNAs were included in TNBC tissues (Fig. 1E). And the cluster analysis of SE-lncRNA expression spectrum of BRCA patients by unsupervised t-SNE algorithm (https://github.com/jkrijthe/Rtsne) revealed that only the SE-lncRNAs in TNBC tissues were significantly different from other subtypes (Fig. 1F). The above results suggest that SE-lncRNA may play an important role in the development of TNBC.

Further analysis of all SE-lncRNAs in relation to TNBC prognosis revealed that 152 SE-lncRNAs were significantly correlated with prognosis (Fig. 1G), of which 23 SE-lncRNAs were differentially expressed in TNBC tissues compared with non-TNBC tissues (Fig. 1H). Machine learning algorithms [30] were widely applied to the exploration of biological big data, 8 characteristic SE-lncRNAs were further screened out using the LASSO algorithm [31] (Fig. 1I), and a TNBC prognostic score model was constructed using the random forest algorithm (https://CRAN.R-project.org/doc/Rnews/), in which the risk score was negatively correlated with the survival time (Fig. 1L), and the patients with high scores had a significantly poorer prognosis (Fig. 1M). The model was evaluated by the ROC curve and compared to the traditional regression model, and the AUC for five-year survival prediction reached 0.938 (Fig. 1J, K). The 8 characteristic SE-lncRNAs were ranked according to their importance (Fig. 1N), with CTB-193M12.5, AP000473.6, and RP11-54O7.17 in the top three. These results suggest that SE-lncRNA expression differentiates BRCA subtypes, and predicts the prognosis of TNBC patients.

### SE-lncRNA RP11-54O7.17 with L2b structural element is down-regulated in TNBC and associated with prognosis

**Fig. 2:**
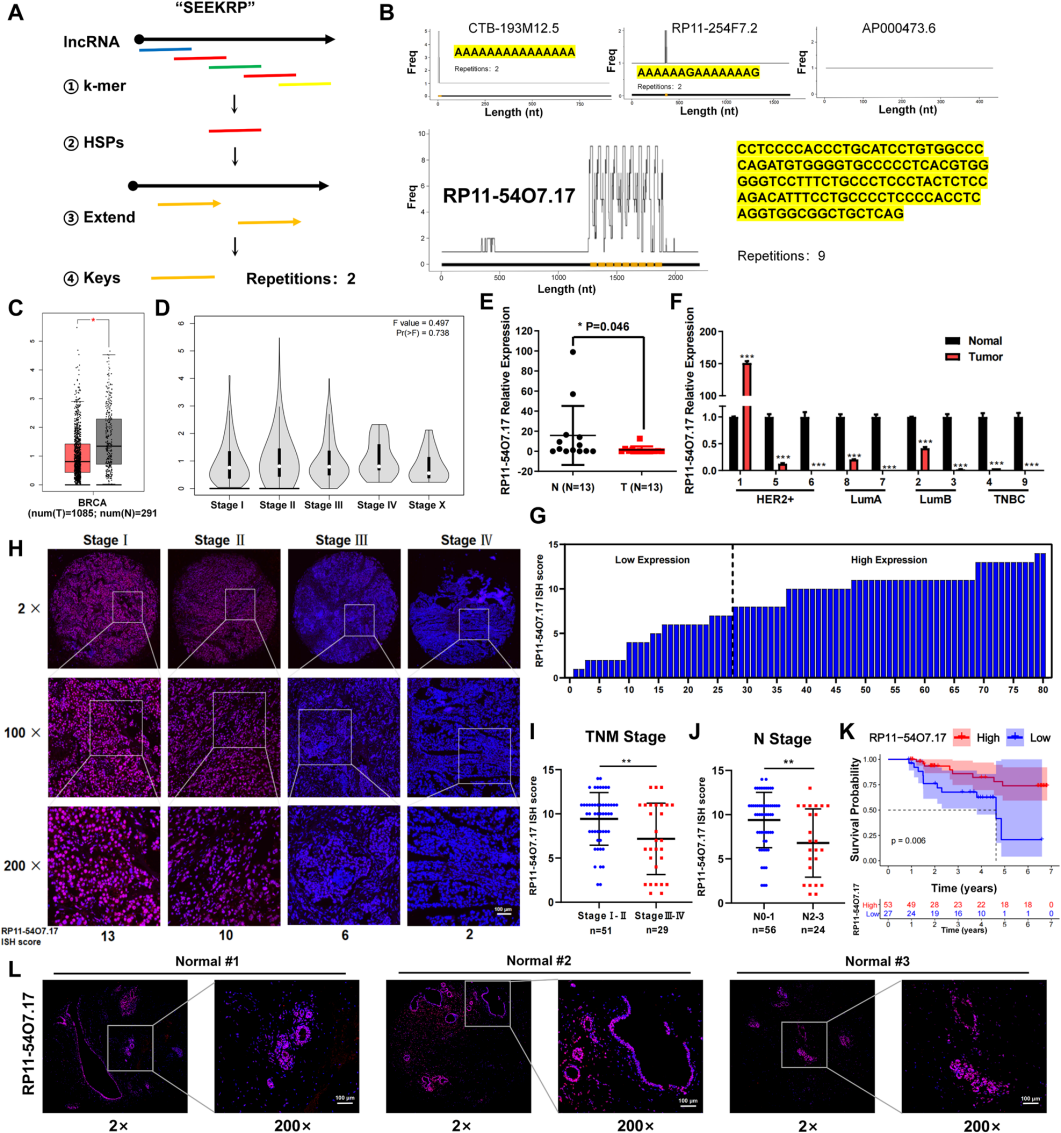
SE-lncRNA RP11-54O7.17 with L2b structural element is down-regulated in TNBC and associated with prognosis. **A** Flowchart of SEEKRP algorithm. **B** Detection of functional fragments of screened genes using SEEKRP algorithm. **C** Expression of RP11-54O7.17 in BRCA cells (red) and normal cells (gray) in the TCGA database. **D** Expression of RP11-54O7.17 in patients with different stages of BRCA in the TCGA database. **E** Expression of RP11-54O7.17 in cancer and paracancerous tissues from paired clinical samples of BRCA patients, *n* = 13. **F** Expression of RP11-54O7.17 in cancer and paracancerous tissues from paired clinical samples of different types BRCA patients, *n* = 9. **G** ISH staining score of 80 TNBC tissues, with ISH scores <7 indicating low expression and ≥7 indicating high expression, *n* = 80. **H**, **I** Distribution map of RP11-54O7.17 ISH staining score points in 80 TNBC tissues with different TNM stages, *n* = 80. **J** In situ hybridization maps and RP11-54O7.17 ISH staining scores for different N stages of TNBC tissues, *n* = 80. **K** Risk score-based survival analysis. TNBC patients with low-expression of RP11-54O7.17 showed a shorter overall survival time than those with high-expression. Overall survival was significantly correlated with TNM stage and lymph node metastasis, but not with age. **L** In situ hybridization maps for RP11-54O7.17 expression in normal breast tissues, *n* = 8. Data for (**E**, **F**, **I** and **J**) are presented as mean ± SD, *P < 0.05, **P < 0.01, ***P < 0.001.

To search for SE-lncRNAs with potential biological functions, the SEEKRP algorithm (Fig. 2A) based on the combination of Blast and SEEKR was developed to identify SE-lncRNAs containing RIDLs (repeat insertion structural domains of lncRNAs). RIDLs are the presence of novel functionalized fragments on some lncRNAs that may originate from transposable element, and these structures may determine the cellular localization and biological functions of lncRNAs. The results showed that the valid repetitive fragment structure was present only in RP11-54O7.17 among the pre-screened SE-lncRNAs (Fig. 2B), and the RepeatMarker 4.1.7 software identification revealed that this fragment belongs to the L2b structural element (Supplementary Fig. 1), which may possess potential biological functions. Other SE-lncRNAs (Fig. 2B) contain common ploy(A) elements (CTB-193M12.5, RP11-254F7.2) or without RIDL (AP000473.6). Subsequently, SE-lncRNA RP11-54O7.17 was selected for the study subject, and a search of the TCGA database showed that it was low-expressed in BRCA tissues (Fig. 2C) and highly absent in advanced BRCA patients (Fig. 2D).

Further RT-qPCR measurement of 9 pairs of clinical samples from different types BRCA and paracancerous tissues revealed that RP11-54O7.17 was commonly low-expressed in BRCA tissues (Fig. 2E, F). Meanwhile, 80 TNBC tissue microarrays were examined by in situ hybridization experiments, and the positive staining intensity values and positive staining cell rates were evaluated by ISH scores, with ISH scores <7 indicating low expression and ≥7 indicating high expression (Fig. 2G). The results showed that the expression of RP11-54O7.17 was significantly negatively correlated with TNM stage and N stage, and its ISH score decreased continuously with increasing stage (Fig. 2H–J). Kaplan–Meier survival curves showed that patients with low-expression of RP11-54O7.17, N2-3 stage and TNM stage III/IV had a shorter overall survival than those with high-expression of RP11-54O7.17, N0-1 stage and TNM stage I/II (Fig. 2K and Supplementary Fig. 2), and RP11-54O7.17 was observed in normal breast tissues with high expression (Fig. 2L). Collectively, these results suggest that RP11-54O7.17 with L2b structural element is downregulated in TNBC and associated with prognosis.

### RP11-54O7.17 is low-expression in TNBC epithelial cells and suppresses the proliferation and metastasis of TNBC cells

Single-cell transcriptome data from 26 BRCA tissues in the GEO database (https://www.ncbi.nlm.nih.gov/gds/?term=GSE176078) were annotated to explore the causes and implications of the low-expression of RP11-54O7.17 in TNBC tissues. The variation of single-cell copy number profiles were estimated by InferCNV algorithm (https://github.com/broadinstitute/inferCNV/wiki) to distinguish tumor cells from normal epithelial cells (Supplementary Fig. 3A). Results showed that RP11-54O7.17 was less expressed in cancerous epithelial cells (Supplementary Fig. 3B), extracted epithelial cells were re-clustered and found that normal epithelial cells were clearly differentiated from cancerous epithelial cells (Supplementary Fig. 3C), and normal epithelial cells in different subtypes of BRCA tissues shared similar gene expression (Supplementary Fig. 3D). Analysis of epithelial cell’s intrinsic subtypes using scSubtype [32] revealed high expression of RP11-54O7.17 in normal mammary duct cells and low expression in basal-like cancerous epithelial cells (Supplementary Fig. 3E, F), which was consistent with the results observed in the previous clinical samples (Fig. 2H, L). The expression of RP11-54O7.17 in normal breast cell line MCF-10A and TNBC cell lines MDA-MB-231 and MDA-MB-468 was further examined, with consistent results found (Supplementary Fig. 3G). Whereas, detection of RNA stability revealed a significant decrease in the half-life of RP11-54O7.17 in MDA-MB-468 (Supplementary Fig. 3H), which may be responsible for the downregulation of RP11-54O7.17 in TNBC cells.

**Fig. 3:**
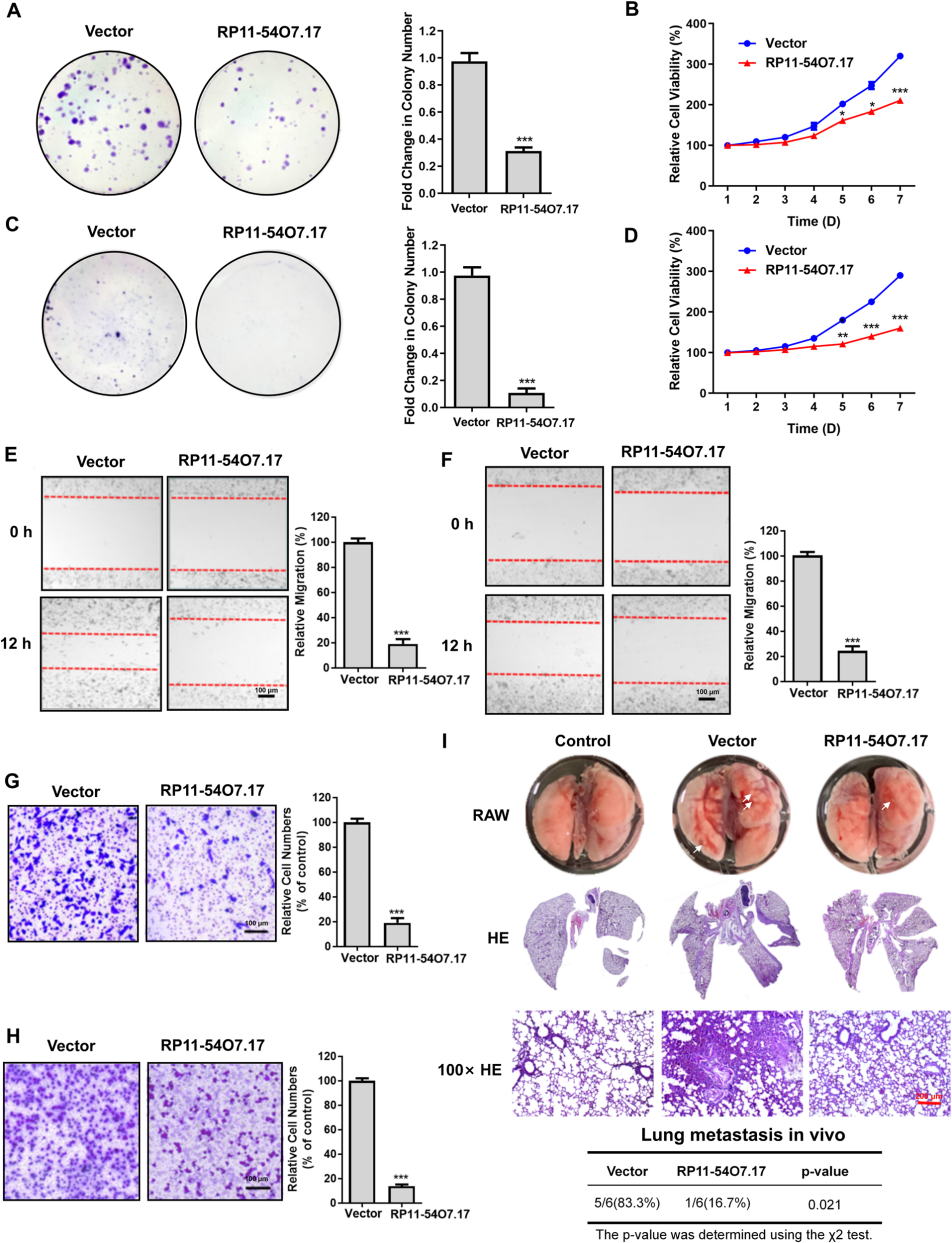
Overexpression of RP11-54O7.17 suppresses TNBC cells proliferation and metastasis. Suppression of MDA-MB-231 proliferation by RP11-54O7.17 overexpression detected by colony formation assay (**A**) and MTT assay (**B**), *n* = 3. Suppression of MDA-MB-468 proliferation by RP11-54O7.17 overexpression detected by colony formation assay (**C**) and MTT assay (**D**), *n* = 3. Suppression of TNBC cells MDA-MB-231 (**E**) and MDA-MB-468 (**F**) migration by RP11-54O7.17 overexpression detected by wound healing assay, *n* = 3. Suppression of TNBC cells MDA-MB-231 (**G**) and MDA-MB-468 (**H**) invasion by RP11-54O7.17 overexpression detected by Transwell assay, *n* = 3. **I** Suppression of tumor lung metastasis in nude mice by RP11-54O7.17 overexpression detected by H&E staining, *n* = 6. Data for (**A**–**H**) are presented as mean ± SD, *P < 0.05, **P < 0.01, ***P < 0.001.

Establishing RP11-54O7.17 overexpressed TNBC cell lines (Supplementary Fig. 4A, B), colony formation assay and MTT assay revealed that overexpression of RP11-54O7.17 significantly inhibited the proliferation of both TNBC cell lines (Fig. 3A–D). Wound healing assay and Transwell assay showed that overexpression of RP11-54O7.177 significantly inhibited the migration and invasion (Fig. 3E–H). MDA-MB-231 cells overexpressing RP11-54O7.17 were injected by tail vein to establish nude mice lung metastasis model. H&E staining revealed that the lung metastasis rate in model group reached 83.3% and only 16.7% in the overexpression of RP11-54O7.17 group, showing that overexpression of RP11-54O7.17 significantly inhibited lung metastasis of TNBC tumors in nude mice (Fig. 3I).

The above results indicates that the expression and stability of RP11-54O7.17 are reduced in TNBC cells compared with these in normal breast duct cells, and that RP11-54O7.17 suppresses the proliferation and metastasis of TNBC cells.

### RP11-54O7.17 suppresses STAT3 activation and down-regulates the expression of downstream genes of SEs

**Fig. 4:**
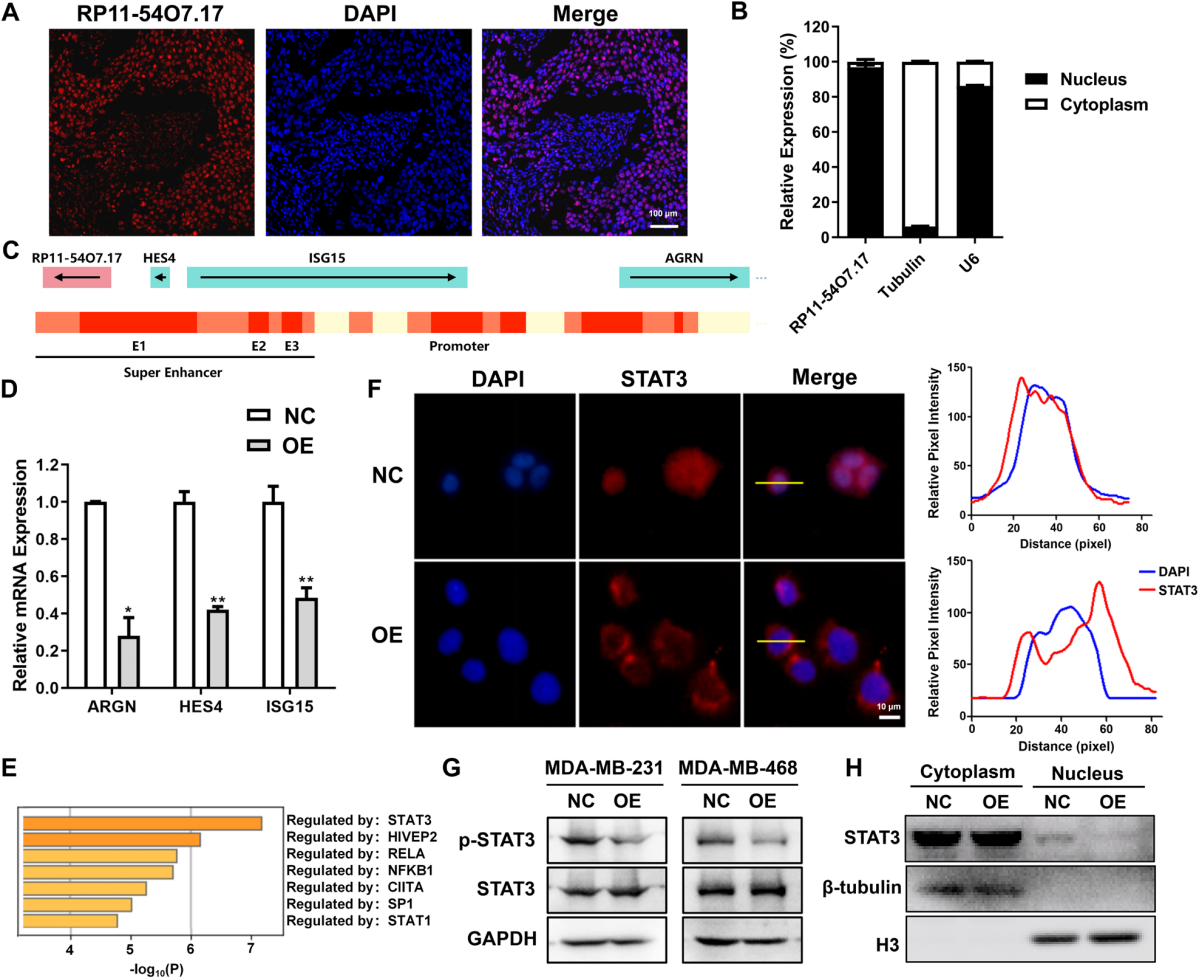
Overexpression of RP11-54O7.17 suppresses genes downstream of SEs and STAT3 activation. **A** Detection of RP11-54O7.17 subcellular localization by in situ hybridization, *n* = 3. **B** Subcellular localization of RP11-54O7.17 by nucleocytoplasmic separation assay, *n* = 3. **C** Schematic representation of related genes downstream of the SE region where RP11-54O7.17 is located. **D** Changes in SE-related downstream genes after RP11-54O7.17 overexpression detected by RT-qPCR assay, *n* = 3. **E** Analysis of transcription factors related to RP11-54O7.17 expression. **F** Effect of RP11-54O7.17 overexpression on STAT3 cellular localization detected by immunofluorescence, *n* = 3. **G** Expression of p-STAT3 and STAT3 in RP11-54O7.17 overexpression TNBC cells detected by Western Blotting, *n* = 3. **H** Intranuclear and extranuclear STAT3 content in RP11-54O7.17 overexpressing cells detected by nucleocytoplasmic separation assay, *n* = 3. Data for (**B** and **D**) are presented as mean ± SD, *P < 0.05, **P < 0.01.

The role and mechanism of lncRNAs are closely related to their subcellular localization [33], thus the subcellular localization of RP11-54O7.17 was examined to investigate the mechanism of RP11-54O7.17 against TNBC. The results of in situ hybridization discovered that RP11-54O7.17 was mainly found in the nucleus (Fig. 4A), which was also verified by subsequent nucleocytoplasmic separation experiments (Fig. 4B), suggesting that RP11-54O7.17 may bind to DNA or proteins in the nucleus for its biological functions.

Several studies have reported that SE-lncRNAs are able to promote the expression of downstream genes of SEs in which these genes located by binding to DNA to form an R-loop [25, 34]. The lncRNA encoded by the human RP11-54O7.17 gene located at 1p36.33 with 2086 nt in length, was searched via the GeneCards database (https://www.genecards.org/), and related downstream genes of the SE in which RP11-54O7.17 located including HES4, ISG15, and ARGN (Fig. 4C). However, overexpression of RP11-54O7.17 was found to generally decrease the transcript levels of these genes using RT-qPCR (Fig. 4D). It has been found that the three genes HES4 [35], ISG15 [36], and ARGN [37] were significant promote the development of TNBC.

SE activation is closely correlated with transcription factors. Transcription factors closely correlated with RP11-54O7.17 were analyzed by SCENIC and found that STAT3 showed the strongest correlation (Fig. 4E). STAT3, a classical cancer marker and target, is overexpressed and constitutively activated in TNBC cells [8]. Phosphorylated STAT3 forms homodimer translocating from the cytoplasm to the nucleus, promoting SE activation [7] and regulating the expression of its downstream target genes, which are involved in cell survival, proliferation, migration, and invasion [10]. Study have shown that the deficiency of STAT3 suppressed the activation of SEs [38]. The effect of RP11-54O7.17 overexpression on STAT3 was examined, and the results showed that RP11-54O7.17 failed to affect the protein expression of STAT3, but suppressed p-STAT3 (Fig. 4G) and blocked the entry of STAT3 into the nucleus (Fig. 4F, H). These results suggest that RP11-54O7.17 suppresses STAT3 activation and downregulates the expression of downstream genes of SEs.

### RP11-54O7.17 binds to S100A4 and modulates autophagy

**Fig. 5:**
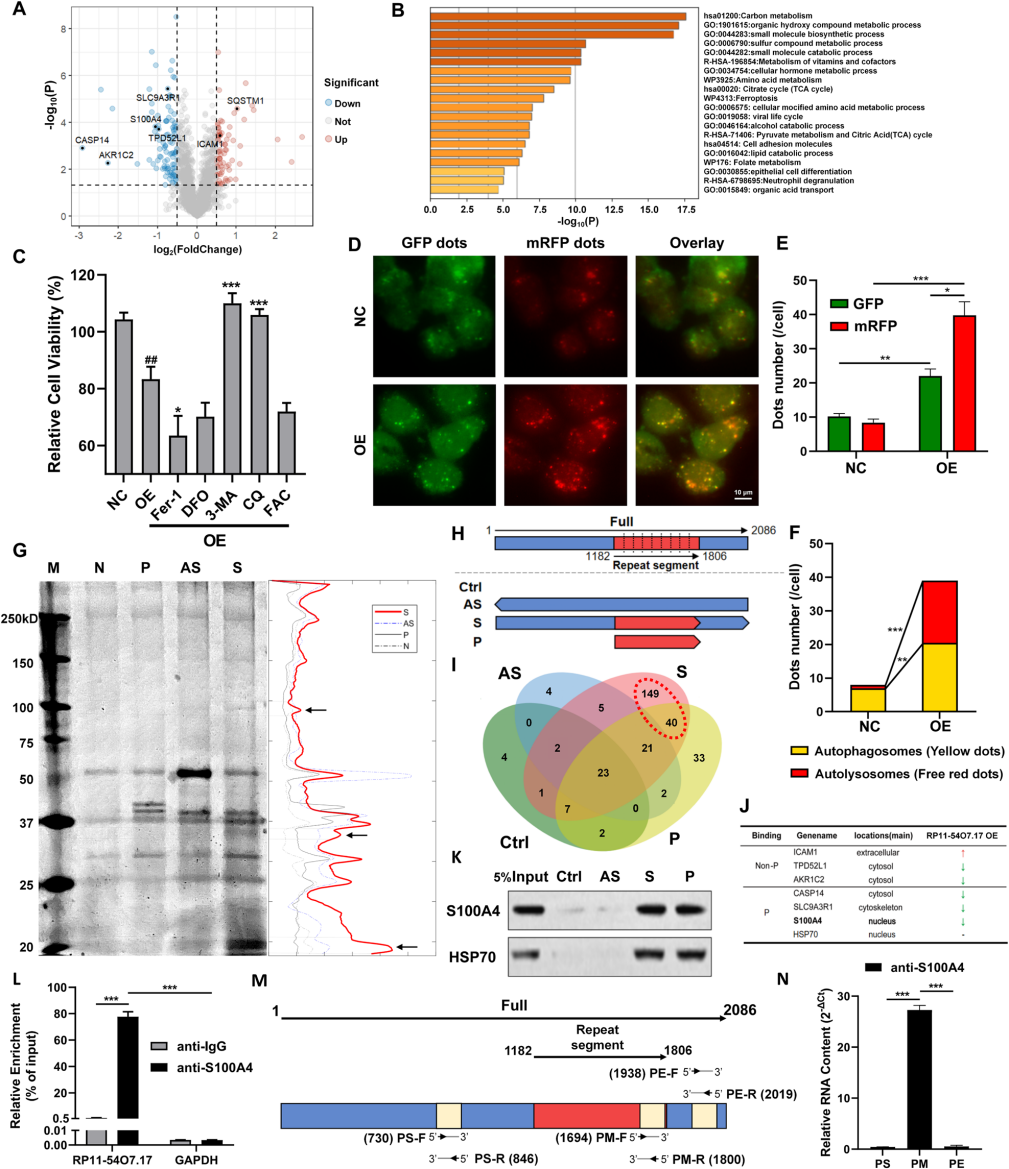
RP11-54O7.17 binds to S100A4 and modulates autophagy. **A** Volcano map of the differential genes after RP11-54O7.17 overexpressed. **B** Proteomic detection of RP11-54O7.17-associated pathways. **C** Effects of autophagy inhibitors (3-MA, CQ), ferroptosis inhibitors (Fer-1, DFO) and ferroptosis inducer (FAC) on the viability of RP11-54O7.17 overexpressed cells, *n* = 3. **D** Representative images of mRFP-GFP-LC3 staining in mRFP-GFP-LC3 transfected into MDA-MB-468 cells, yellow spots in the merged image represent autophagosomes and red represents autolysosome, *n* = 3. **E** Statistics on the intensity of red and green fluorescence of mRFP-GFP-LC3, *n* = 3. **F** Statistics on the number of autophagosomes and autolysosomes in RP11-54O7.17 overexpressed cells, *n* = 3. **G** RNA pull down assay for RP11-54O7.17 binding proteins. Left is the silver staining of RNA pull down and right is the gray scale analysis of silver staining (M, marker. N, negative control. P, positive control. AS, antisense. S, sense), *n* = 3. **H** Schematic diagram of the grouping of RP11-54O7.17 binding proteins detected by RNA pull down-proteomics coupled assay (Ctrl, negative control. AS, antisense. S, sense. P, repeat part). **I** Veen diagram of different subgroups of RP11-54O7.17 binding proteins. **J** Statistics of RP11-54O7.17 binding proteins and differential proteins induced by RP11-54O7.17 overexpressed (Non-P, combined in non-repetitive fragments. P, combined in repeating fragments. Red up arrow, protein content increased after RP11-54O7.17 overexpressed. Green down arrow, protein content decreased after RP11-54O7.17 overexpressed. Horizontal bar, no significant change in protein content after RP11-54O7.17 overexpressed). **K** Bindings of S100A4 and HSP70 to RP11-54O7.17 were verified by RNA pull down combined with Western blotting, *n* = 3. **L** Binding of RP11-54O7.17 to S100A4 detected by RIP assay, *n* = 3. **M** Schematic representation of RP11-54O7.17 primers for different fragments. **N** Bindings of RP11-54O7.17 fragments to S100A4 detected by crosslinking RIP assay, *n* = 3. Data for (**C**, **E**, **L** and **N**) are presented as mean ± SD, *P < 0.05, **P < 0.01, ***P < 0.001, ^##^ *P* < 0.01.

Proteomics and RNA Pull Down assays were used to further explore the specific molecular mechanism by which RP11-54O7.17 regulates STAT3 to suppress TNBC. The differential proteins of before and after RP11-54O7.17 overexpression were detected using proteomics (Fig. 5A, Supplementary Table 1) and enriched using Metascape (https://metascape.org), which revealed that a series of metabolism-related pathways and ferroptosis were enriched (Fig. 5B), while autophagy-related proteins, SQSTM1 (P62) showed significant differential changes. Pre-treatment of autophagy inhibitors 3-MA and CQ significantly reversed the proliferation inhibitory activity of RP11-54O7.17 whereas the ferroptosis inhibitors Fer-1 and DFO as well as the ferroptosis inducer FAC failed to effect (Fig. 5C). The effect of RP11-54O7.17 overexpression on autophagic flux was examined using GFP-mRFP-LC3 lentiviral stably transfected MDA-MB-468 cells, and found that the overexpression significantly promoted the LC3B protein level and the formation of intracellular autophagosomes and autolysosomes (Fig. 5D–F).

LncRNAs in the nucleus are able to serve functions by binding to proteins or DNA [39, 40]. Previous studies found that RP11-54O7.17 failed to upregulate the downstream genes of SE, but inhibited STAT3 activation (Fig. 4D, E, H). It is therefore hypothesized that RP11-54O7.17 may exert its biological function via binding to proteins. It was found that RP11-54O7.17 was able to specifically bind to a few proteins with molecular weights in the range of 10–20 KDa by streptomycin-labeled RNA pull-down and silver-staining assays (Fig. 5G). The protein-binding ability of the repeat fragment in RP11-54O7.17 was verified by RNA pull-down experiments combined with mass spectrometry detection of the binding of control, antisense, repeat and full-length sequences to the protein (Fig. 5H). The results showed that RP11-54O7.17 was able to bind to 189 proteins, of which 40 bound to the repeat fragment (Fig. 5I). The proteins which bound specifically to RP11-54O7.17 and changed significantly during RP11-54O7.17 overexpression were counted. Based on the results, ICAM1, TPD52L1, AKR1C2, CASP14, SLC9A3R1, S100A4, six proteins were identified as meeting the screening criteria and the latter three were bound to repeat fragment (Fig. 5J). Other proteins bound to RP11-54O7.17 but with insignificant changes, including HSP70, which binds to the repeat fragment, may function through molecular chaperones (Supplementary Table 2). Considering the subcellular localization, it was found that only S100A4 with a molecular weight of 11.4 KDa was mainly localized in the nucleus [41]. It has been reported that S100A4 activates STAT3 in cancer cells [42], while proteomics results showed that overexpression of RP11-54O7.17 decreased intracellular S100A4 content. Therefore, it is hypothesized that RP11-54O7.17 exerts its biological functions by regulating S100A4. Taken together, these results indicate that RP11-54O7.17 is able to modulate autophagy while binding to S100A4 via repeat fragments.

### RP11-54O7.17 suppresses TNBC via S100A4/STAT3 signaling axis

**Fig. 6:**
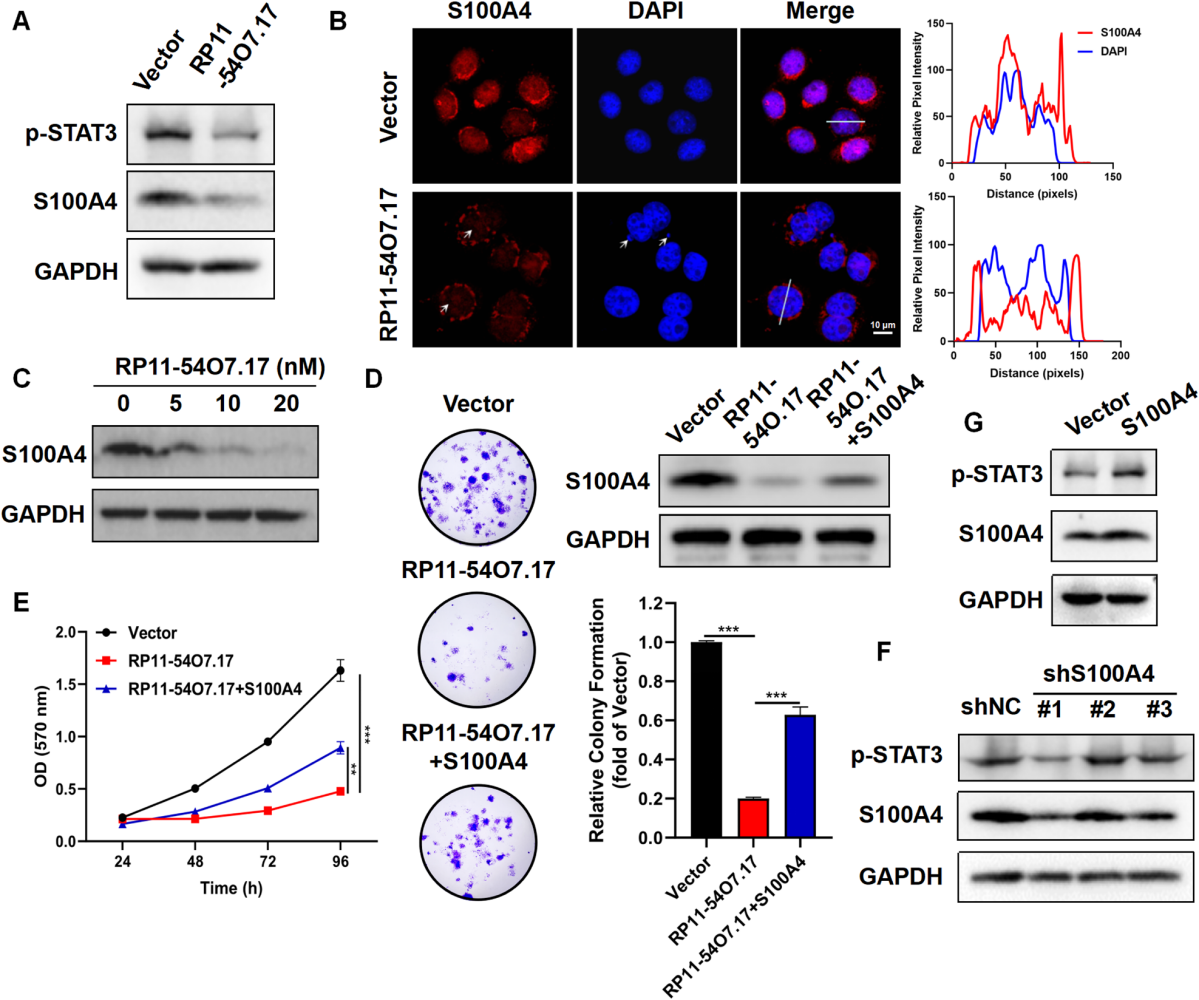
RP11-54O7.17 suppresses the proliferation of MDA-MB-468 cells by S100A4/STAT3 signaling axis. **A** Expression of S100A4 and p-STAT3 in RP11-54O7.17 overexpressed cells detected by Western blotting, *n* = 3. **B** Expression of S100A4 in RP11-54O7.17 overexpressed cells detected by immunofluorescence (White arrows: S100A4 nucleoli enrichment, DAPI micronuclei), *n* = 3. **C** Expression of S100A4 in cells transfected with different concentrations of RP11-54O7.17 in vitro, *n* = 3. **D** Effect of S100A4 overexpression on the inhibition of cell clone formation induced by RP11-54O7.17 by colony formation assay, *n* = 3. **E** Effect of S100A4 overexpression on the inhibition of cell viability induced by RP11-54O7.17 by MTT assay, *n* = 3. **F** Effect of S100A4 knocked-down on STAT3 activation, n = 3. **G** Effect of S100A4 overexpression on STAT3 activation, *n* = 3. Data for (**D**, **E**) are presented as mean ± SD, **P < 0.01, ***P < 0.001.

To verify whether RP11-54O7.17 exerts a role in modulating TNBC through the suppression of STAT3 mediating by S100A4, Western blotting was used to examine the impact of RP11-54O7.17 overexpression on the S100A4/STAT3 signaling axis in TNBC cells, which revealed that the overexpression decreased the expression of S100A4 and p-STAT3 in TNBC cells (Fig. 6A). Immunofluorescence assay results showed that S100A4 was mainly localized in the nucleus and more in the nucleolus and nuclear membrane, and the S100A4 content in RP11-54O7.17-overexpressed nuclei was significantly decreased, along with the production of micronuclei (Fig. 6B). Transfection of RP11-54O7.17 suppressed the viability of TNBC cells in a dose-dependent manner (Supplementary Fig. 5A, B), while decreased S100A4 expression (Fig. 6C). S100A4-overexpressed and -knockdown MDA-MB-468 cell lines were constructed and verified by S100A4 mRNA and protein assays (Supplementary Fig. 6, Fig. 6F, G), the colony formation and MTT assays revealed that S100A4 overexpression reduced the impact of RP11-54O7.17 on colony formation and cell viability of TNBC cells (Fig. 6D, E). These results suggest that RP11-54O7.17 modulates TNBC by regulating S100A4.

Meanwhile, S100A4 knockdown suppressed STAT3 activation in TNBC cells (Fig. 6F), while S100A4 overexpression promoted STAT3 activation (Fig. 6G). STAT3-knocked-down TNBC cell lines were constructed by shRNA and found that STAT3 knockdown eliminated the inhibitory effect of RP11-54O7.17 overexpression on TNBC cell proliferation (Supplementary Fig. 5C, D). The above results indicate that RP11-54O7.17 suppresses TNBC cell proliferation by modulating the S100A4/STAT3 signaling axis.

### RP11-54O7.17 promotes autophagy-lysosome degradation of S100A4

**Fig. 7:**
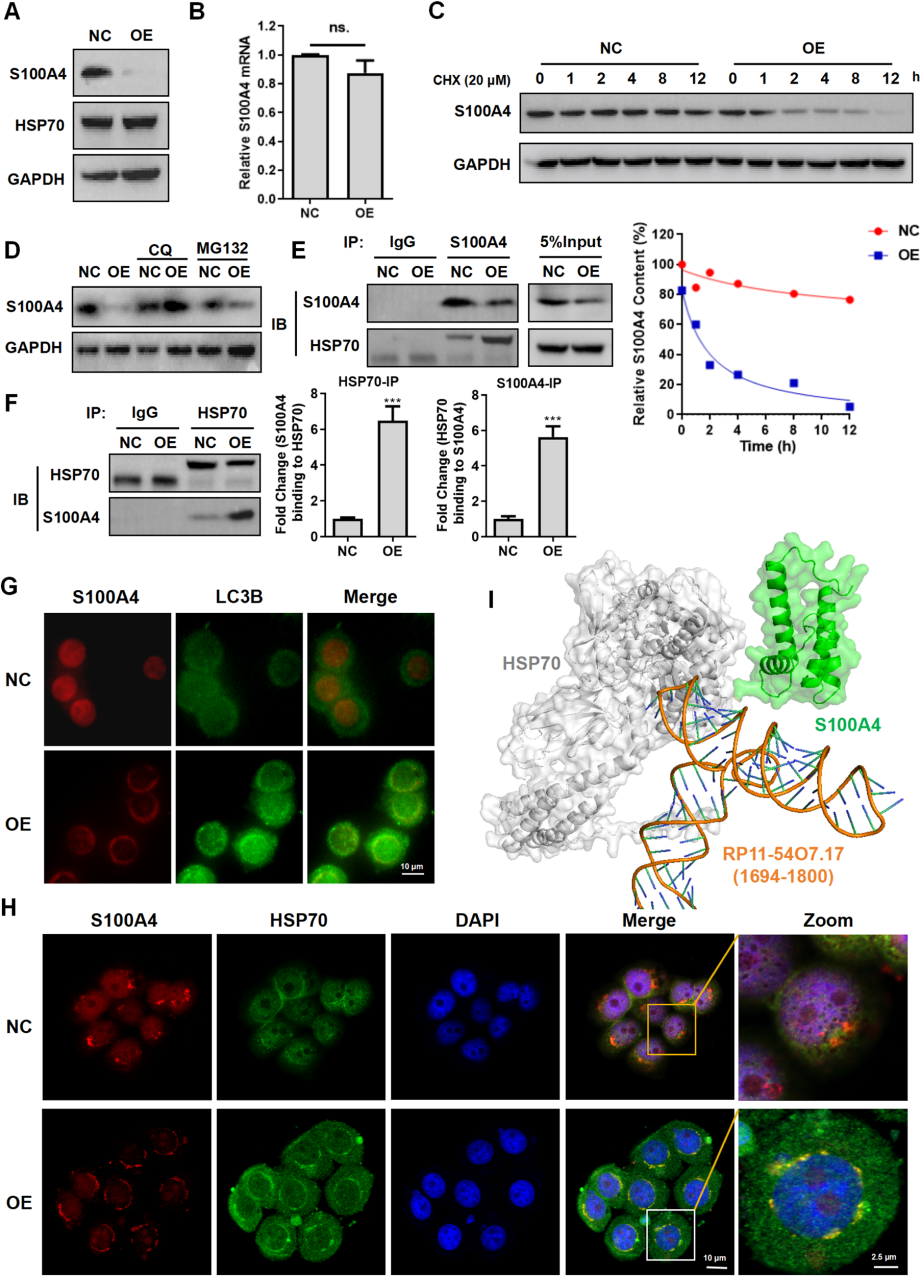
RP11-54O7.17 promotes autophagy-lysosome degradation of S100A4 through the binding of repeat fragment P to S100A4. **A** Effect of RP11-54O7.17 overexpression on stability of S100A4 protein detected by Western blotting, and gray scale analysis, *n* = 3. **B** Effect of RP11-54O7.17 overexpression on mRNA level of S100A4 detected by RT-qPCR, *n* = 3. **C** Effect of autophagy inhibitor CQ and proteasome inhibitor MG132 on S100A4 protein content in RP11-54O7.17 overexpressed cells, *n* = 3. **D** Effect of S100A4 translocation toward autolysosomes in RP11-54O7.17 overexpressed MDA-MB-468 cells detected by immunofluorescence, *n* = 3. **E** Expression of S100A4 and HSP70 in RP11-54O7.17 overexpressed MDA-MB-468 cells, *n* = 3. **F**, **G** Effect of RP11-54O7.17 overexpression on the interaction of S100A4 with HSP70 detected by Co-IP, and gray scale analysis, *n* = 3. **H** Effect of RP11-54O7.17 overexpression on co-localization of S100A4 and HSP70 detected by immunofluorescence, *n* = 3. **I** Complex structure of S100A4/HSP70/ RP11-54O7.17 (fragment) simulated by Alphafold3. Data for (**B**, **G**) are presented as mean ± SD, ***P < 0.001.

To further explore the mechanism by which RP11-54O7.17 downregulates S100A4, the impact of RP11-54O7.17 on the stability of S100A4 was determined. Compared with control cells, S100A4 degradation was faster in RP11-54O7.17 overexpressed cells (Fig. 7A), while RP11-54O7.17 overexpression did not affect the mRNA level of S100A4 (Fig. 7B). In addition, the autolysosome inhibitor CQ was able to significantly reverse the downregulation of S100A4 induced by RP11-54O7.17, whereas the ubiquitin proteasome inhibitor MG-132 exerted a weak effect (Fig. 7C). Immunofluorescence assays showed that RP11-54O7.17 overexpression promoted the translocation of S100A4 to autolysosomes (Fig. 7D). These results suggest that RP11-54O7.17 affects S100A4 expression through the autophagy-lysosome pathway.

Further studies revealed that RP11-54O7.17 and the repeat fragment P therein were able to bind to S100A4 and HSP70 (Fig. 5K). Reverse validation by RIP experiments showed that S100A4 was able to specifically bind to RP11-54O7.17 (Fig. 5L, Supplementary Fig. 7A, B) and the binding site was located at repeat fragment P (Fig. 5M, N, Supplementary Fig. 7C, D). S100A4 expression was decreased in RP11-54O7.17 overexpressed cells, while HSP70 was not significantly affected (Fig. 7E).

It has been reported that HSP70 is able to bind to RNA [43] and promote protein degradation by the autophagy-lysosome pathway [44], and the pre-experiments revealed that RP11-54O7.17 was able to bind to HSP70 (Fig. 5J, K). It was hypothesized that RP11-54O7.17 may promote S100A4 degradation by constructing the S100A4/HSP70 complex. Immunoprecipitation results showed that RP11-54O7.17 overexpression promoted the interaction of S100A4 with HSP70 (Fig. 7F, G). Immunofluorescence results showed that RP11-54O7.17 overexpression promoted the co-localization of HSP70 and S100A4 (Fig. 7H). The complex structure of S100A4/HSP70/RP11-54O7.17 (fragment) was simulated using Alphafold3 (Fig. 7I). Immunofluorescence results showed that RP11-54O7.17 overexpression promoted the co-localization of HSP70 and S100A4 (Fig. 7K). In summary, these results indicate that RP11-54O7.17 promotes autophagy-lysosome degradation of S100A4 by constructing the S100A4/HSP70 complex.

### RP11-54O7.17 liposome suppresses the growth of TNBC in vivo

A large number of studies have found that lncRNAs have important roles in cancer [45–47], however the development of drugs targeting lncRNAs has mainly focused on techniques such as RNA interference to target carcinogenic lncRNAs, while the development of anticarcinogenic lncRNAs has been minimal.

**Fig. 8:**
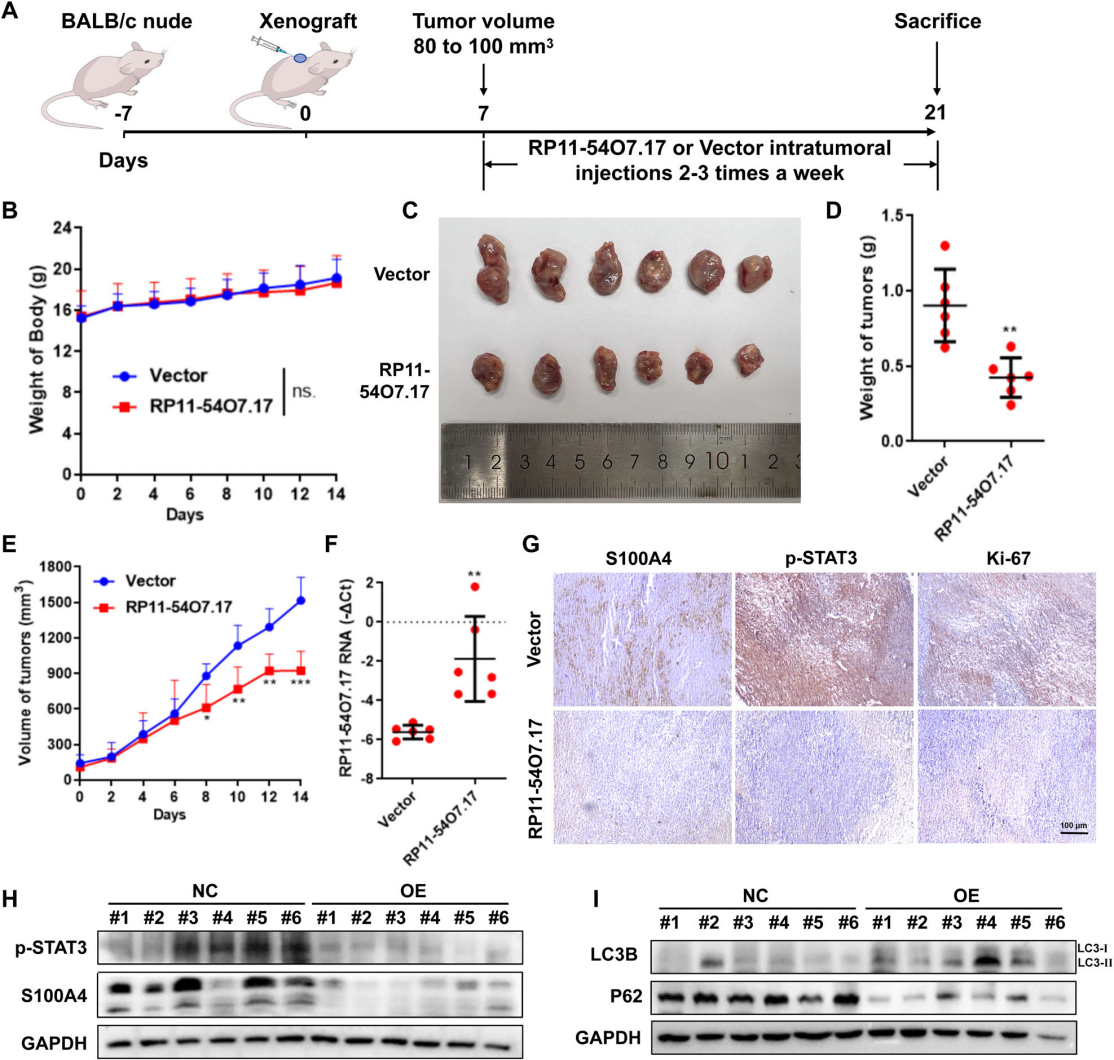
RP11-54O7.17 liposome suppresses TNBC growth in vivo. **A** RP11-54O7.17 Liposome delivery modality. **B** Changes in nude mice body weight after RP11-54O7.17 liposome injection, *n* = 6. **C** Images of subcutaneous tumors, *n* = 6. **D** Weight of subcutaneous tumors, *n* = 6. **E** Changes in subcutaneous tumor volume, *n* = 6. **F** Content of RP11-54O7.17 tumor tissue, *n* = 6. **G** Expression of S100A4, p-STAT3 and Ki-67 in tumor tissues detected by immunohistochemistry, *n* = 3. **H** Expression of p-STAT3 and S100A4 in tumor tissues detected by Western blotting, *n* = 3. **I** Expression of LC3B and P62 in tumor tissues detected by Western blotting, *n* = 3. Data for (**B**, **D**, **E**, and **F**) are presented as mean ± SD, *P < 0.05, **P < 0.01, ***P < 0.001.

The above results have shown that RP11-54O7.17 was able to anti-TNBC proliferation in vitro at a high concentration, so further RP11-54O7.17 liposome was prepared and examined for its in vivo inhibitory activity against TNBC by intratumoral injection (Fig. 8A). The results showed that RP11-54O7.17 liposome suppressed TNBC growth in vivo (Fig. 8C–E) without significant systemic toxicity (Fig. 8B). This approach effectively increased the content of RP11-54O7.17 in tumor tissues (Fig. 8F). Immunohistochemical results were consistent with in vitro, and elevated RP11-54O7.17 significantly reduced the protein content of S100A4, p-STAT3 and Ki-67 in tumor tissues (Fig. 8G) and suppressed the expression of p-STAT3 and S100A4 (Fig. 8H). Meanwhile, Western blotting revealed a significant activation of the autophagy-lysosome pathway in the tumor tissues (Fig. 8I). In conclusion, RP11-54O7.17 liposome exhibits favorable anti-TNBC effects without significant systemic toxicity in vivo.

## Discussion

This study found that the expression spectrum of SE-lncRNA in TNBC was significantly different from that of non-TNBC, and the constructed random forest-based scoring model was able to predict the prognosis of patients. Clinical samples showed that the key SE-lncRNA RP11-54O7.17 was low-expressed in TNBC tissues and correlated with poor prognosis, while low-expression was observed in patients with high TNM stage and metastases. Single-cell transcriptome and RT-qPCR results showed that the level and stability of RP11-54O7.17 in TNBC cells were much lower, while overexpression of RP11-54O7.17 in vitro and in vivo was able to suppress the proliferation and metastasis of TNBC cells. Analysis of the base sequences revealed that the L2b-type repetitive structural element in RP11-54O7.17 might be the key fragment for its biological functions. It was further found that RP11-54O7.17 directly interacted with S100A4 precisely through this key fragment to promote the binding of S100A4 to the molecular chaperone HSP70, leading to the autophagy-lysosome degradation of S100A4, and thus suppressing the phosphorylation and nuclear translocation of STAT3 against TNBC. Moreover, RP11-54O7.17 liposomes also exerted favorable anti-TNBC effects in vivo. Altogether, these results demonstrate that RP11-54O7.17 is a potential target for TNBC treatment.

Nucleic acid drugs such as small interfering RNAs, microRNAs, nucleic acid aptamers, and small activating RNAs developed on short-stranded noncoding RNAs have been widely approved for cancer treatment and achieved great therapeutic efficacy [48–50]. As an essential class of non-coding RNAs, lncRNAs and their functional fragments have not been developed into cancer therapeutic drugs yet, despite their proven therapeutic effects on cancer [40]. SEs play key roles in maintaining cancer cell identity and promoting oncogenic transcription [8, 51], our group had reported that inhibition of SEs-related proteins RARα and CDK7 exerts anti-TNBC effects [9]. Activation of SEs is accompanied by the production of corresponding lncRNAs, which represent highly potential therapeutic targets for TNBC, and the exploration of mechanisms and drug-forming possibilities of SE-lncRNAs will bring novel promises for TNBC treatment.

Structural modularity is a special property shared by the secondary and tertiary structures of lncRNAs. Despite the rapid evolution of the primary structure of lncRNAs, modular fragments are highly conserved, which determines the unique function of modular fragments to bind to RNAs, DNAs, or proteins and to exert biological roles [52]. A number of literatures demonstrating that transposable elements have contributed repeatedly and profoundly to the evolution of genome structure and function through the insertion of preformed sequence elements [53]. To identify SE-lncRNAs with drug potential, machine learning and SEEKRP algorithms were used to screen out the anti-TNBC lncRNA RP11-54O7.17, which contains a repetitive sequence.

Single-cell transcriptome data annotated by scSubtype to understand the mechanism by which RP11-54O7.17 modulates TNBC proliferation and metastasis revealed that RP11-54O7.17 expression was significantly decreased in basal-type carcinoma cells compared with normal epithelial cells, with consistent results obtained in normal breast cells and TNBC cells. In vitro and in vivo overexpression of RP11-54O7.17 significantly suppressed the proliferation and metastasis of TNBC. Meanwhile, the relatively poor stability of RP11-54O7.17 in TNBC cells might be the reason for its decreased content. It has been reported that SE-lncRNA may enhance the expression of SEs downstream genes by promoting the production of intranuclear R-loop [34, 54]. In situ hybridization and nucleocytoplasmic separation assays showed that RP11-54O7.17 was mainly localized in the nucleus, while unlike previously reported SE-lncRNAs, the assay revealed that RP11-54O7.17 overexpression significantly suppressed the expression of downstream genes of SEs which the RP11-54O7.17 located. We therefore hypothesized that RP11-54O7.17 suppresses the SE, whose activity is closely related to transcription factor activation. Transcription factor correlation analysis revealed that STAT3 was the transcription factor most closely related to RP11-54O7.17, whereas RP11-54O7.17 overexpression was able to suppress STAT3 phosphorylation and nuclear translocation. Proteomic analysis of RP11-54O7.17 overexpressed TNBC cells showed significant differential changes in autophagy- and ferroptosis-related proteins, but only autophagy-associated inhibitors exhibited reversal of RP11-54O7.17 anti-TNBC effects.

lncRNA-protein complexes play an important role in the development of various diseases [55]. The results of RNA pull-down along with proteomics assays showed that 40 proteins directly bound to RP11-54O7.17 and its repeat fragments, of which only S100A4 was mainly localized in the nucleus and decreased in RP11-54O7.17 overexpressed cells. It has been reported that S100A4 in TNBC cells is able to activate STAT3 [25]. The levels of p-STAT3 and S100A4 were significantly decreased in RP11-54O7.17 overexpressed cells, whereas carcinoma cell viability and S100A4 expression were dose-dependently reduced after transcription of different concentrations of RP11-54O7.17 in vitro. Further results revealed that the autolysosome inhibitor of protein degradation pathway significantly reversed the downregulation of S100A4, while S100A4 overexpression reversed the inhibitory activity of RP11-54O7.17 on TNBC cells. Furthermore, once STAT3 was knocked-down, the effects of RP11-54O7.17 on TNBC cell proliferation and metastasis were significantly attenuated. In this study, HSP70 was found to be able to bind to the RP11-54O7.17 repeat fragment without significant expression changes. It has been reported that as a molecular chaperone, HSP70 is able to bind to target proteins and then transport to the lysosome [56], as well as bind to RNA [43], so it was hypothesized that RP11-54O7.17 connected the HSP70 to S100A4. The results showed that the interaction and co-localization of HSP70 with S100A4 was significantly enhanced in RP11-54O7.17 overexpressed cells, and the simulation results of RP11-54O7.17/S100A4/HSP70 complex by AlphaFold3 provided support for this hypothesis. Taken together, RP11-54O7.17 is likely to promote autophagy-lysosome degradation of S100A4 by constructing the S100A4/HSP70 complex.

Currently, TNBC is experiencing a poor patient prognosis due to a lack of effective targeted therapies, the discovery of RP11-54O7.17 provides novel perspectives on the treatment of TNBC. This study demonstrates that it may be novel therapeutic strategies to restore the expression of RP11-54O7.17 or directly using RP11-54O7.17 liposomes to intervene in TNBC cells. In addition, the expression and activity of RP11-54O7.17 may serve as a biomarker for prognostic assessment of TNBC, which may help clinicians to develop a more personalized treatment plan. Further studies could focus on exploring the synergistic effects of RP11-54O7.17 with existing TNBC treatments to enhance therapeutic efficacy. However, there are still some issues that need to be addressed. The specific mechanism of RP11-54O7.17 in the development of TNBC needs to be further characterized. For instance, it is necessary to confirm the specific structural binding sequence pattern of the RP11-54O7.17/S10A4/HSP70 complex to promote the efficient development of lncRNA nucleic acid drugs. RP11-54O7.17 was also found to be differentially expressed in different macrophages in TNBC, and S100A4 was also found to play a role in tumor immunity, thus the interaction of RP11-54O7.17 with other signaling pathways and its role in the tumor microenvironment need to be further explored. In addition, the clinical potential of RP11-54O7.17 liposomes needs to be validated by more preclinical and clinical studies.

**Fig. 9:**
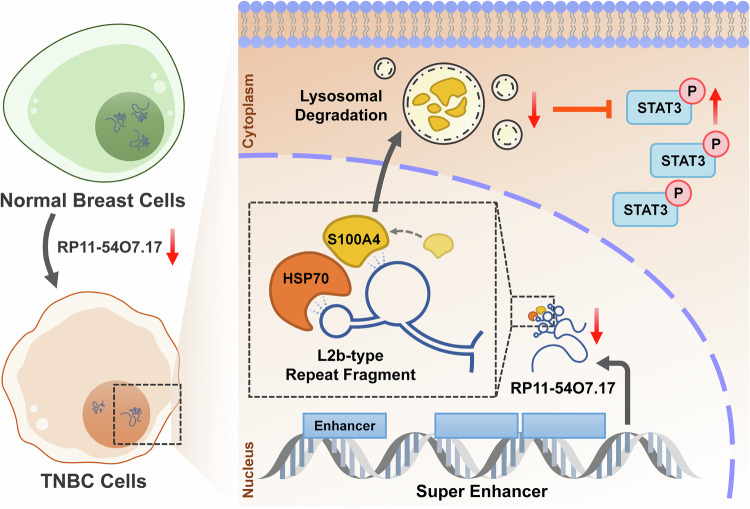
Mechanism of action of RP11-54O7.17 mediated TNBC. RP11-54O7.17 is low-expressed and destabilized in TNBC cells, and the RP11-54O7.17 overexpression enhances the interaction of S100A4 with HSP70, promoting the autophagy-lysosome degradation of S100A4, which in turn suppresses the transcription of STAT3 downstream oncogenes.

In summary, our study demonstrates that RP11-54O7.17 is low-expressed and destabilized in TNBC cells. Mechanistically, we identify the RP11-54O7.17/S100A4-STAT3 signaling axis in TNBC, where the RP11-54O7.17 overexpression enhances the interaction of S100A4 with HSP70, promoting the autophagy-lysosome degradation of S100A4, which in turn suppresses the transcription of STAT3 downstream oncogenes (Fig. 9). The therapeutic effects of RP11-54O7.17 liposomes in vivo suggest that RP11-54O7.17 serves as a potential target and drug precursor for treatment of TNBC.

## Methods

### Ethics statement

The studies were approved by the Ethics Committee of the Ruian People’s Hospital of Wenzhou Medical University (No. LunKZ20211028D). We informed all related patients of the use of these specimens and got the written informed consent. The study using the tissue microarray was approved by the Life Sciences Ethics Committee of Changsha Yaxiang Biotechnology Co., LTD, and the ethics report is available online at https://yxswll.ccrl.cn/, which the query code is YK9BHBG5JDN6PP. The animal experiment was approved by the Institutional Animal Care and Use Committee of Zhejiang Chinese Medical University (No. 20241225-01) and were performed in accordance with the guidelines proposed by the Laboratory Animal Research Center of Zhejiang Chinese Medical University.

### Cell culture

TNBC cell lines MDA-MB-231, MDA-MB-468 and human normal mammary epithelial cell line MCF-10A obtained from the National Collection of Authenticated Cell Cultures (Identification through STR, Shanghai, China). All cell lines were cultured in DMEM supplemented with 10% fetal bovine serum (FBS, Sijiqing, Hangzhou, China) at 37 °C in 5% CO_2_. The medium was replaced every other day, and cells were passaged every two days.

### Colony formation assay

MDA-MB-231, MDA-MB-468 cells were digested and collected, and inoculated into 6-well plates at 1 × 10^3^ cells per well. After being placed in a 37 °C, 5% CO_2_ incubator for 14 d, the cells were fixed using 4% paraformaldehyde for 25 min, then stained with 0.1% crystal violet staining solution. ImageJ software was used to calculate the number of colonies.

### Cell proliferation assay

The cells collected above were inoculated into 96-well plates at 4 × 10^3^ cells per well. After being placed in a 37 °C, 5% CO_2_ incubator for 48 h, 20 μL of MTT reagent (5 mg/mL) was added to each well and incubation was continued for 4 h. Subsequently, formazan crystals were completely dissolved using dimethyl sulfoxide, the absorbance at 490 nm of each well was measured.

### Wound healing assay

The cells collected above were inoculated into 24-well plates at 2 × 10^5^ cells per well and cultured to confluence. Uniform scratches were scraped in monolayer cells and recordings were taken at 0 and 12 h to assess the migration level.

### Transwell invasion assay

The cells collected above were resuspended using serum-free medium, and inoculated with 1 × 10^5^ cells in the upper chamber of a Transwell chamber containing matrigel, then 600 μL of complete medium containing 10% FBS was added to the lower chamber. After 12 h of culture, cells in Transwell chamber where invasion occurred were fixed using 100% methanol and then stained with 0.1% crystal violet staining solution. ImageJ software was used to calculate the number of invasive cells.

### Preparation of plasmid expression vector and shRNA

RP11-54O7.17 (RP11-54O7.17 cDNA were cloned into the lentivirus vector GV513) were obtained from GeneChem (Shanghai, China). S100A4 (S100A4 cDNA were cloned into the lentivirus vector pENTER) were obtained from Weizhen (Shandong, China). ShRNAs for STAT3 designed by GeneChem were derived from our previous related research [57]. ShRNAs for S100A4 (S100A4 shRNAs were cloned into the lentivirus vector pLent-U6-CMV-copGFP-P2A-Puro) were obtained from Weizhen. Plasmids, shRNAs, or lncRNAs were transfected into TNBC cells with Lipofectamine 2000 (Invitrogen). RP11-54O7.17 were transfected into cells for RP11-54O7.17 overexpression. S100A4 were transfected into cells for S100A4 overexpression. STAT3-shRNA were transfected into cells for STAT3 knockdown. S100A4-shRNA were transfected into cells for S100A4 knockdown. Vectors were transfected into cells for negative control. The relevant shRNA sequences were presented in the Supplementary Table 3.

### Reverse transcription and quantitative real-time PCR (RT-qPCR)

The total RNA in the samples was extracted using Trizol and cDNA was synthesized using the Evo M-MLV reverse transcription kit (Accurate, Hunan, China). Real-time quantitative PCR was subsequently performed using the SYBR Green Pro Taq HS premixed qPCR kit (Accurate, Hunan, China). The relative RNA expression was determined by the 2^-ΔΔCt^ method. PCR primer sequences were shown in the Supplementary Table 4.

### LncRNA and liposome preparation

To synthesize and amplify RP11-54O7.17 cDNA, PCR was conducted using forward primers containing the T7 RNA polymerase promoter sequence. PCR products were purified according to the instruction of DNA Gel Extraction Kit (Beyotime, China). RP11-54O7.17 lncRNA was synthesized by T7 RNA Polymerase Kit (Beyotime, China) according to the instruction. Liposomes of RP11-54O7.17 were prepared with Lipofectamine 2000 (Invitrogen). The relevant T7-PCR-Primer sequences were presented in the Supplementary Table 5.

### RNA stability assay

TNBC cells and MCF-10A cells were administrated with actinomycin D (5 μg/mL) at 2, 4, 6, 8 h, respectively, and collected at 10 h. Then the total RNA was extracted and analyzed by RT-qPCR.

### Western blotting

TNBC cells were collected and lysed by RIPA lysate containing phosphatase inhibitor and protease inhibitor. Equal masses of proteins were separated by SDS-PAGE gel electrophoresis and transferred to PVDF membranes. After incubation with 5% nonfat milk, membranes were incubated with primary antibodies and further incubated with HRP-conjugated secondary antibody. Protein complexes were detected by ECL. Primary antibodies in the study were antibodies against p-STAT3 (1:1000, Cst, #4113), STAT3 (1:1000, Cst, #30835), S100A4 (1:1000, Cst, #13018), HSP70 (1:1000, Santa Cruz, #K2020), GAPDH (1:1000, Cst, #5174), β-tubulin (1:1000, Cst, #2128) and H3 (1:1000, Millipore, #06-599).

### Cytoplasmic and nuclear RNA/protein purification

Nucleoplasmic separation was performed using the nuclear and cytoplasmic protein extraction kit (Beyotime Biotechnology, Shanghai, China). TNBC cells were collected, cytoplasm was extracted using a cytoplasmic extraction reagent, and nuclei were extracted by repeated vigorous vortexing for 30 min using a nuclear extraction reagent. Protein was extracted by RIPA, and RNA was extracted by Trizol, then the RNA and protein expression were detected by Western Blotting and RT-qPCR.

### Immunofluorescence

The cultured TNBC cells were fixed with 4% paraformaldehyde and after permeabilization with 0.5% Triton X-100, then the cells were blocked using 1% BSA. Subsequently, primary antibody was added and incubated at 4 °C overnight, and further incubated with fluorescent secondary antibody at room temperature for 2 h, and nuclei were stained using DAPI. Imaging was performed using a fluorescence microscope.

### Immunoprecipitation

MDA-MB-231, MDA-MB-468 cells overexpressing RP11-54O7.17 or control cells were collected and lysed by NETN lysate buffer containing protease inhibitor. The supernatant of the lysate was collected, and 1/10 of it was added to SDS-PAGE upsampling buffer and boiled as input. Another lysate containing 500 μg of protein was taken and co-incubated with primary antibody or IgG, followed by addition of agarose beads for overnight incubation. The agarose beads-antibody-protein complexes were washed with NETN lysate buffer, and the precipitated proteins were eluted in SDS-PAGE upwelling buffer, then the analysis was performed by western blotting.

### Proteomics analysis

Proteomics analysis of RP11-54O7.17 before and after overexpression was carried out based on Q-Exactive and Orbitrap Exploris 480 mass spectrometry platform, and the proteomic data were analyzed using the Maxquant, Perseus, and Proteome Discoverer software.

### Fluorescent in situ hybridization (FISH)

FISH experiments were performed using Fluorescence in Situ Hybridization Kit for RNA (Beyotime, Shanghai, China), and the RP11-54O7.17 specific probe was synthesized by Servicebio (Wuhan, China). Cell samples were fixed using 4% paraformaldehyde, then digested with 10 μg/mL proteinase K and fixed again. The samples were washed with 0.5 M HCl and acetylated by acetylation solution. Pre-hybridization solution containing yeast RNA was added and incubated at 40 °C for 1 h. The pre-hybridization solution was discarded, and hybridization solution containing the probe was added and hybridized overnight at the same temperature and washed with SSC wash buffer containing formamide deionized. The subcellular localization of RP11-54O7.17 was detected using fluorescence microscopy.

### Silver staining

Silver staining assays were performed using Fast Silver Stain Kit (Beyotime, Shanghai, China). SDS-PAGE gels obtained in Western Blot were fixed with fixative containing 50% ethanol and 10% acetic acid. After sensitization with silver staining sensitizing solution, the gel was incubated with 0.01% silver solution for 10 min and with silver staining color developing solution for 3–10 min. Once the expected bands were clearly visible, the reaction was immediately terminated with silver staining terminating solution.

### RNA pull-down

RNA pull-down experiments were performed using Magnetic RNA-protein Pull-Down Kit (Thermo, MA, USA). RNA-protein complexes were formed by incubating biotin-labeled probes with cell lysates at 30 °C. RNA binding buffer-pretreated streptavidin agarose magnetic beads were incubated at room temperature to precipitate the complexes. The complexes were washed with RNA wash buffer, and the proteins were eluted using SDS-PAGE sampling buffer, then the analysis was performed by western blotting and silver staining assays.

### RNA binding protein immunoprecipitation (RIP)

TNBC cells overexpressing RP11-54O7.17 or control cell samples were cross-linked with or without formaldehyde. After lysing the cells using the NETN lysate buffer mixture containing protease inhibitors and RNase inhibitors, Trizol or SDS-PAGE upsampling buffer was added to the lysate and boiled as input for RT-qPCR or western blotting, respectively. Another lysate was taken and co-incubated with primary antibody or IgG, followed by addition of agarose beads for overnight incubation. The agarose beads-antibody-protein complexes were washed with NETN lysate buffer. Heat to uncrosslink the RNA-protein cross-links of the cross-linked cell samples. Further elute the RNA or protein by Trizol or SDS-PAGE upsampling buffer, then the analyze was performed by RT-qPCR or western blotting.

### CHX-chase assay

Protein half-life was detected using CHX inhibition of protein synthesis. MDA-MB-231, MDA-MB-468 cells overexpressing RP11-54O7.17 or control cells were digested and collected, and inoculated into 6-well plates at 3 × 10^5^ cells per well. Fresh serum-free medium was added with 50 μg/mL CHX at different times and protein expression was detected at 0, 1, 2, 4, 8, and 12 h by western blotting.

### Xenograft

MDA-MB-231 cells were digested and collected, and injected subcutaneously into the back of 4-week-old female BALB/c-nude mice at 3 × 10^6^ cells, then the mice were randomly divided into control and treatment groups using a random number method. Upon reaching a tumor volume of 100 mm^3^, liposome-encapsulated RP11-54O7.17 or 0.9% saline was injected into the mice tumors 2–3 times per week for 14 d. The mice weight and tumor volume were recorded every 2 days. All mice were euthanized at the end of the experiment, and tumors were collected and weighed for preparation of lysates or fixation in 4% paraformaldehyde for analysis, respectively.

### Pulmonary metastatic model

MDA-MB-231 overexpressing RP11-54O7.17 or control cells were digested and collected, and injected into female BALB/c-nude mice via the tail vein at 3 × 10^6^ cells to establish a mouse model of lung metastasis. After 3 weeks, all mice were euthanized, and the lungs were collected and fixed in 4% paraformaldehyde for analysis.

### Statistical analysis

Data were shown as mean ± SD of at least three experiments. Comparison between groups was considered statistically significant when *P* < 0.05 by Tukey’s post hoc test, unpaired *t*-test or chi-squared test, which was conducted using GraphPad Prism 8, SPSS, R and ImageJ software.

## Supplementary information

Supplementary Figures

Supplementary Table legends

Supplementary Table 1

Supplementary Table 2

Supplementary Table 3

Supplementary Table 4

Supplementary Table 5

Full and uncropped western blots

## Data Availability

The authors declare that all relevant data of this study are available within the article or from the corresponding author on reasonable request.
