# Supplementary material for: Super enhancer lncRNA RP11-54O7.17 regulates the proliferation and metastasis of triple-negative breast cancer by targeting lysosomal degradation of S100A4
Source: Cell Death Dis. 2025 Oct 31;16(1):773. doi: 10.1038/s41419-025-08072-3 (PMC12578829; doi:10.1038/s41419-025-08072-3)
Supplement: Supplementary file 1 — Supplementary Figures [file 41419_2025_8072_MOESM1_ESM.docx]

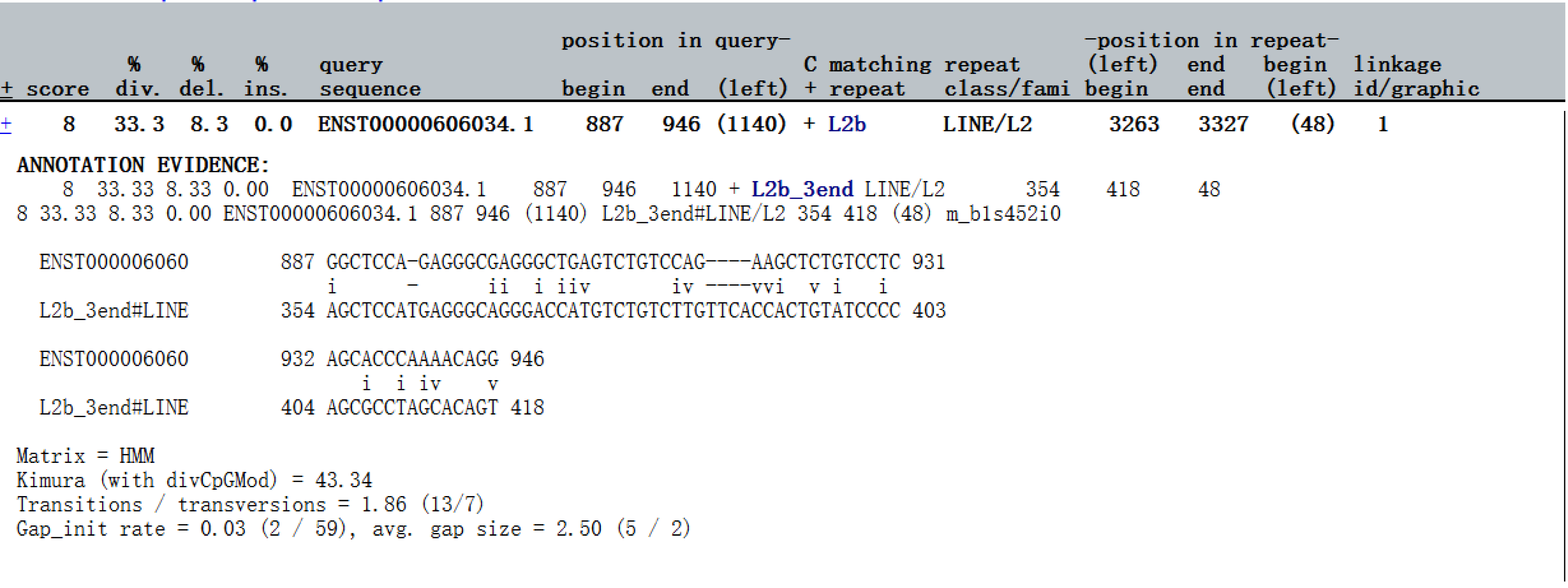


**Supplementary Fig. 1. RepeatMarker 4.1.7 software identifies the repeat fragment of RP11-54O7.17 belongs to the L2b structural element.**


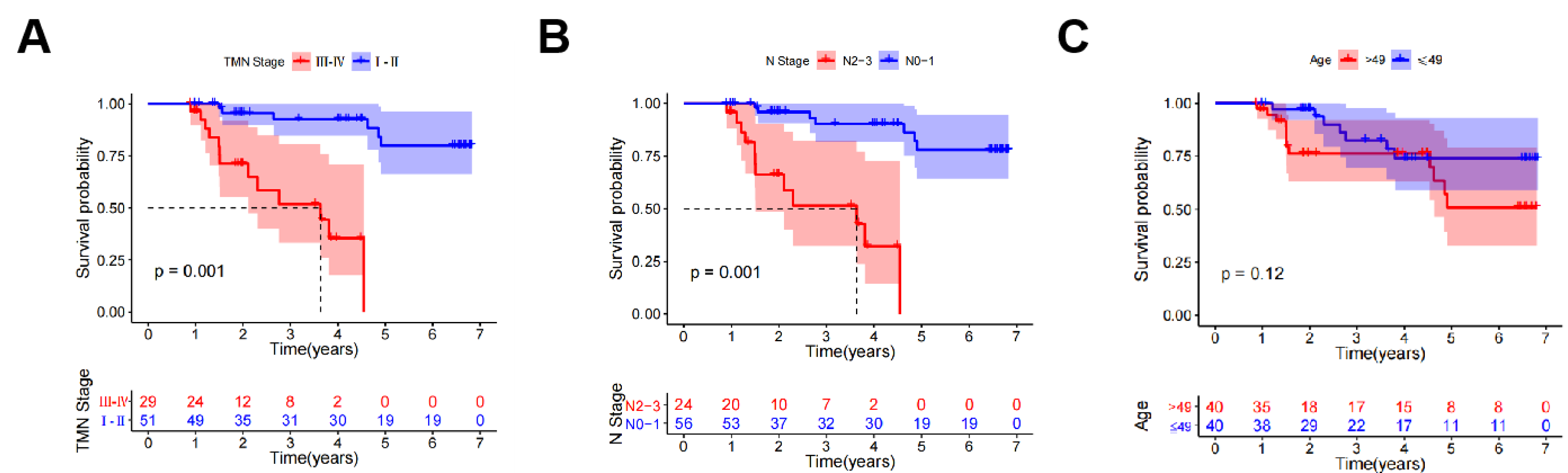


**Supplementary Fig. 2. Analysis of the correlation between clinical characteristics and prognosis of TNBC patients. A**. TNM stage. **B**. N stage. **C**. Age.


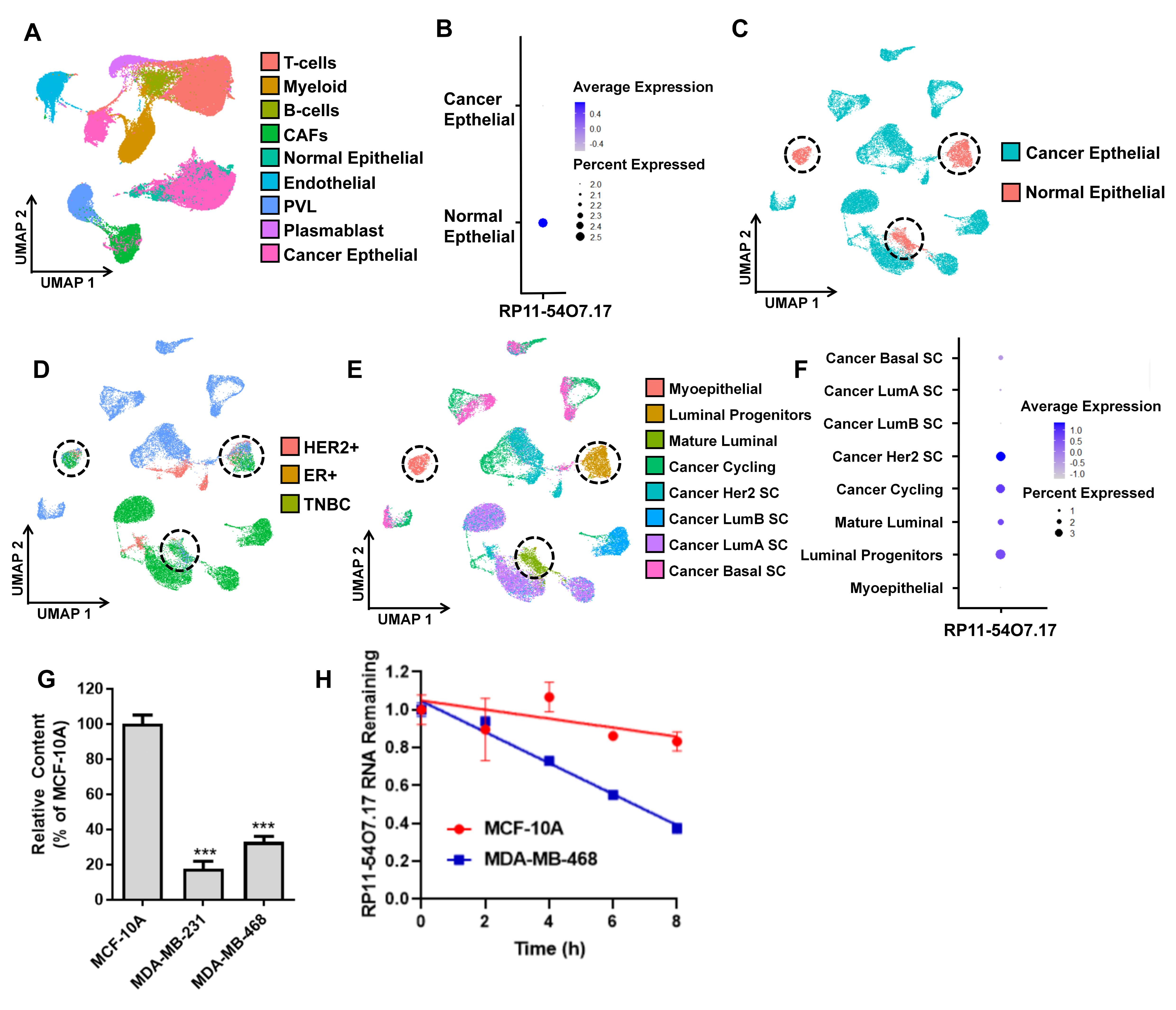


**Supplementary Fig. 3. Low expression and stability of RP11-54O7.17 in TNBC cells. A**. Single-cell transcriptome data (GSE176078) clustering and annotation for BRCA tissues. **B**. RP11-54O7.17 expression in normal and cancerous epithelial cells. **C-E**. UMAP visualization of all epithelial cells, colored by tumor (**C**), clinical subtype (**D**), scSubtype subtype (**E**). **F**. RP11-54O7.17 expression in epithelial cells of different scSubtype subtypes. **G**. RT-qPCR detection of RP11-54O7.17 expression in MCF-10A, MDA-MB-231 and MDA-MB-468 cells, n=3. **H**. Actinomycin D assay for RP11-54O7.17 stability in MCF-10A and MDA-MB-468 cells, n=3. Data for (**G, H**) are presented as mean ± SD, ****P* < 0.001.


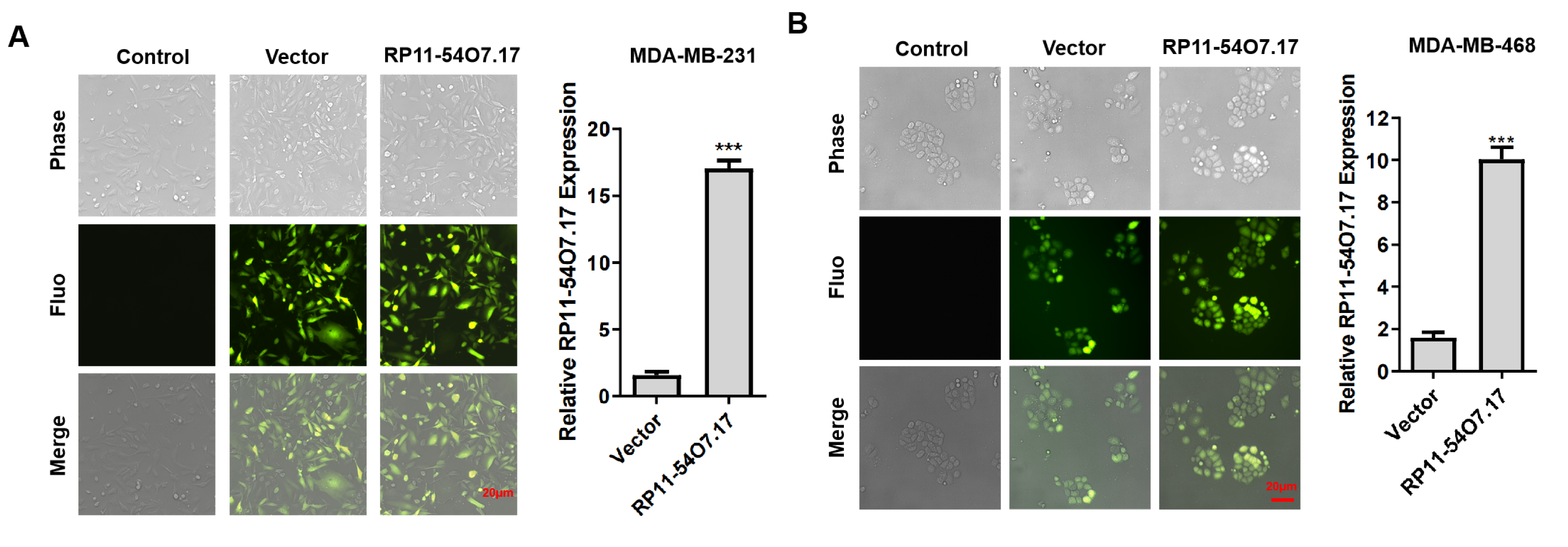


**Supplementary Fig. 4.** **Construction of RP11-54O7.17 overexpressed TNBC cell lines. A.** Fluorescence microscopy of GFP expression in MDA-MB-231 cells to assess transfection, and PCR to detect RP11-54O7.17 content in overexpressed cells. **B.** Fluorescence microscopy of GFP expression in MDA-MB-468 cells to assess transfection, and PCR to detect RP11-54O7.17 content in overexpressed cells. Data for (**A**, **B**) are presented as mean ± SD, ****P* < 0.001.


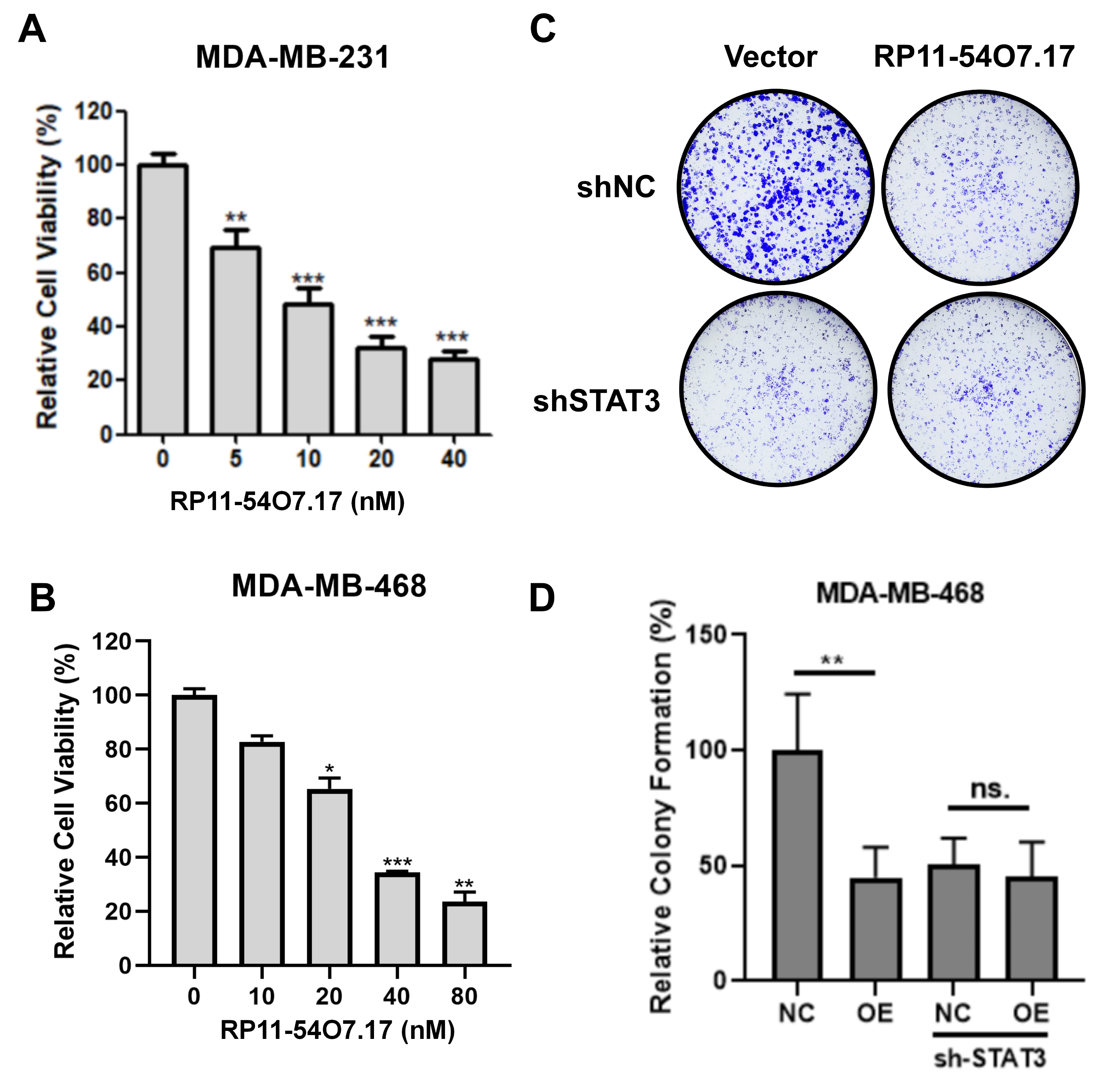


**Supplementary Fig. 5. RP11-54O7.17 regulates TNBC mediated by STAT3**. **A-B**. Viability of MDA-MB-231 (**A**), MDA-MB-468 (**B**) cells at different concentrations of RP11-54O7.17 by MTT assay, n=3. **C-D**. Effect of STAT3 knocked-down on the inhibition of MDA-MB-468 cell clone formation induced by RP11-54O7.17 by colony formation assay (C), and statistical analysis (D), n=3. Data for (**A**, **B**, and **D**) are presented as mean ± SD, **P* < 0.05, ***P* < 0.01, ****P* < 0.001.


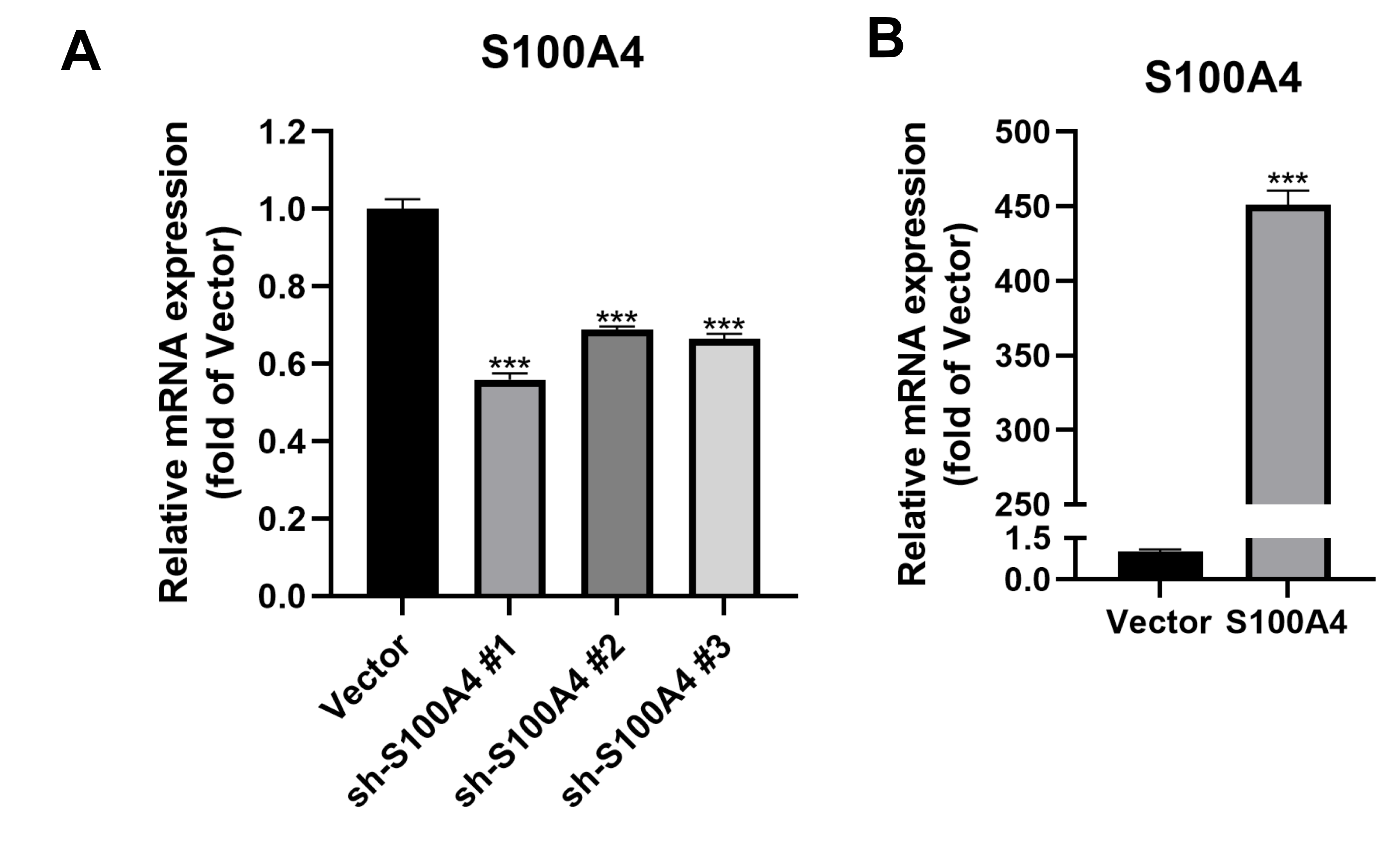


**Supplementary Fig. 6.** The mRNA levels of knockdown (**A**) and overexpression (**B**) of S100A4 were verified by RT-qPCR, n=3. Data are presented as mean ± SD, ****P* < 0.001.


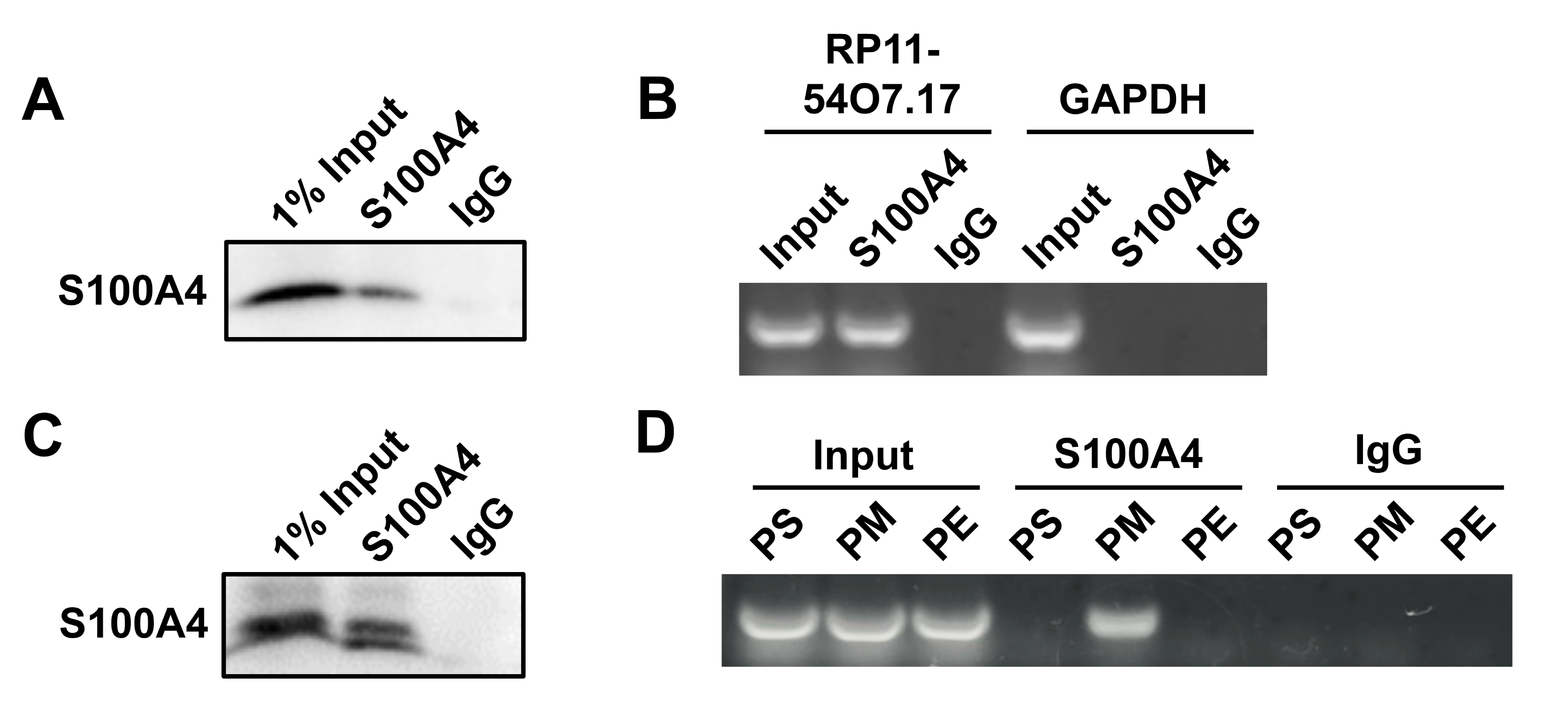


**Supplementary Fig. 7.** Binding fragment of RP11-54O7.17 to S100A4 detected by RIP assay. **A**. Specificity of S100A4 pull-down in RIP assay verified by Western blotting, n=3. **B**. Specific binding of intracellular S100A4 to RP11-54O7.17 verified by RIP assay, n=3. **C**. Specificity of S100A4 pull-down in cross-linked ultrasound RIP assay verified by Western blotting, n=3. **D**. Specific binding of intracellular S100A4 to the repeat fragments in RP11-54O7.17 verified by RIP assay, n=3.
