# Supplementary material for: Super enhancer lncRNA RP11-54O7.17 regulates the proliferation and metastasis of triple-negative breast cancer by targeting lysosomal degradation of S100A4
Source: Cell Death Dis. 2025 Oct 31;16(1):773. doi: 10.1038/s41419-025-08072-3 (PMC12578829; doi:10.1038/s41419-025-08072-3)
Supplement: Supplementary file 2 — Supplementary Table legends [file 41419_2025_8072_MOESM2_ESM.docx]

**Supplementary Table 1. Proteomic profiling of MDA-MB-468 cells after RP11-54O7.17 overexpression.**

**Supplementary Table 2. RNA pull-down assay identifies RP11-54O7.17-interacting proteins.**

**Supplementary Table 3. shRNA sequences for gene knockdown.**

**Supplementary Table 4. Primer sequences for RT-qPCR**

**Supplementary Table 5. T7 promoter primers for in vitro lncRNA synthesis.**
