# Supplementary material for: Super enhancer lncRNA RP11-54O7.17 regulates the proliferation and metastasis of triple-negative breast cancer by targeting lysosomal degradation of S100A4
Source: Cell Death Dis. 2025 Oct 31;16(1):773. doi: 10.1038/s41419-025-08072-3 (PMC12578829; doi:10.1038/s41419-025-08072-3)

Fig. 4 Overexpression of RP11-54O7.17 suppresses genes downstream of SEs and STAT3 activation.

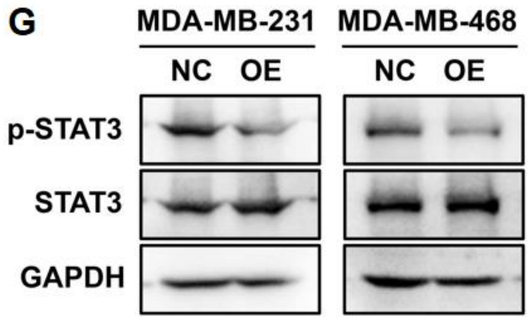

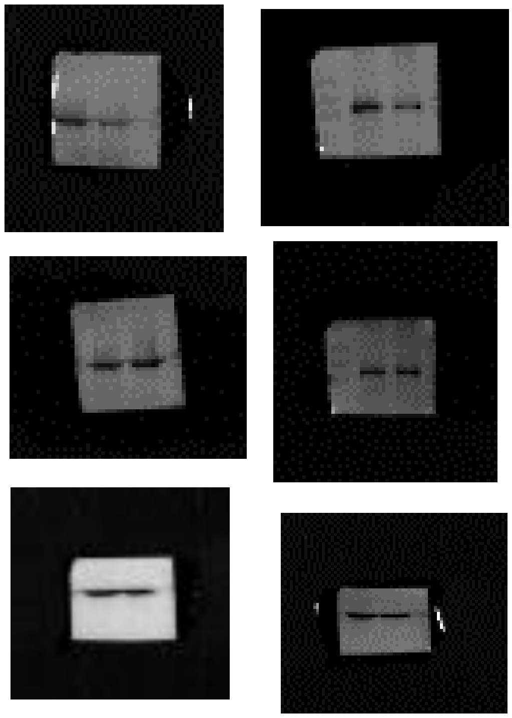

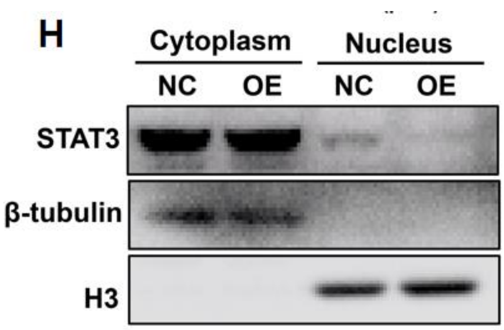

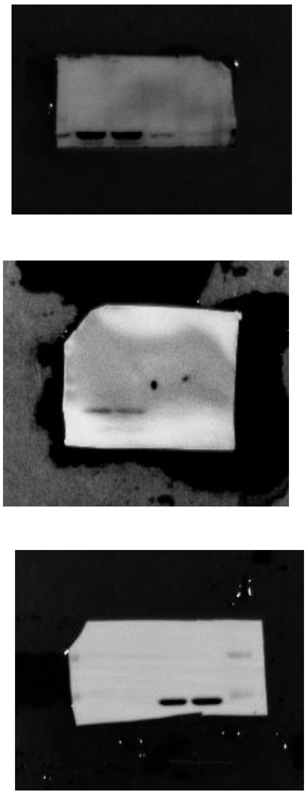

Fig. 6 | RP11-54O7.17 suppresses the proliferation of MDA-MB-468 cells by S100A4/STAT3 signaling axis.

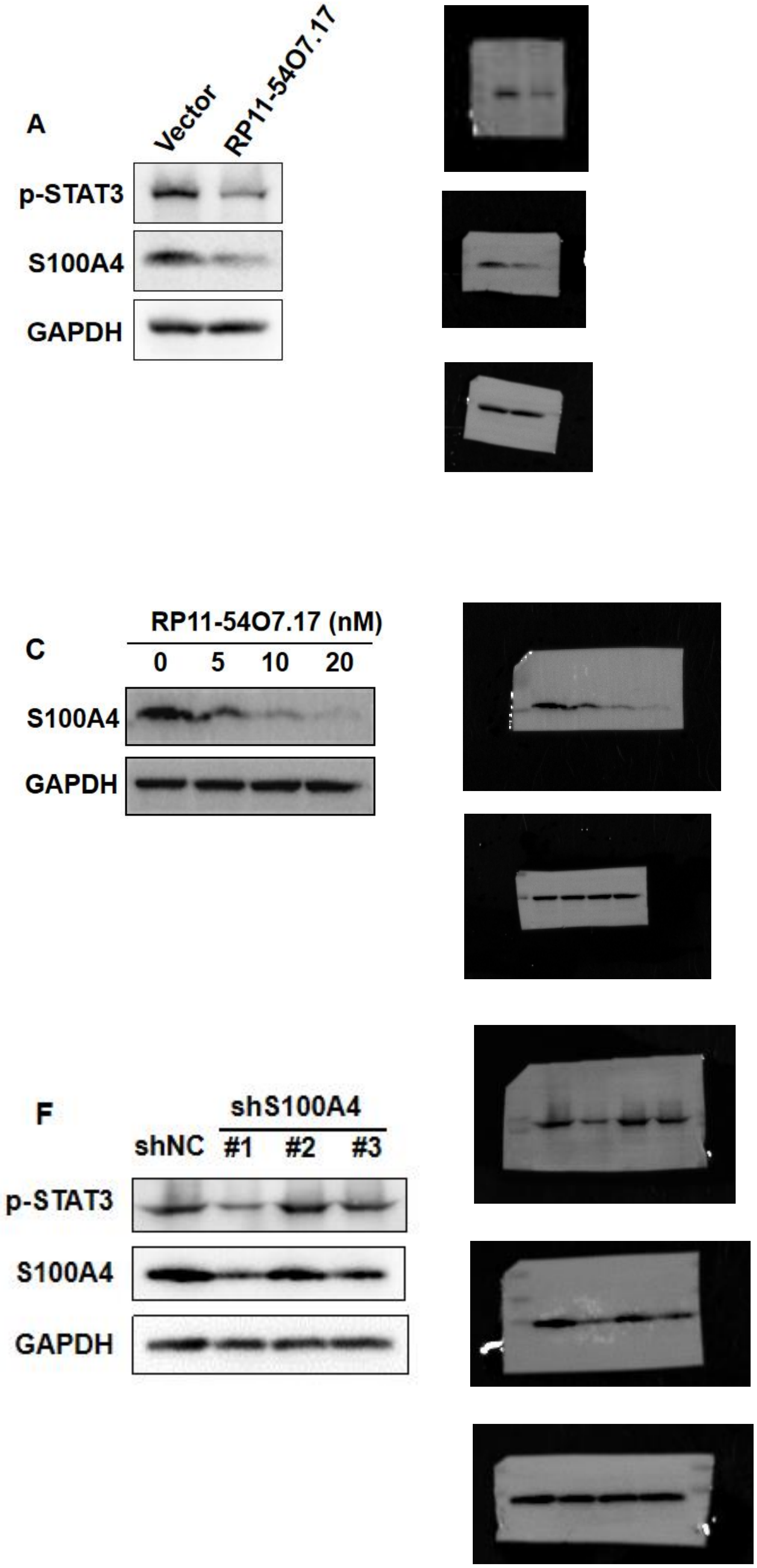

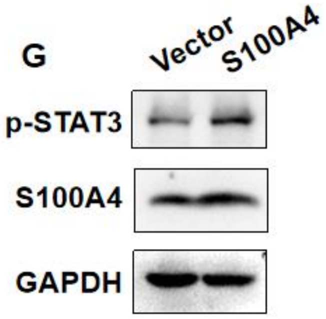

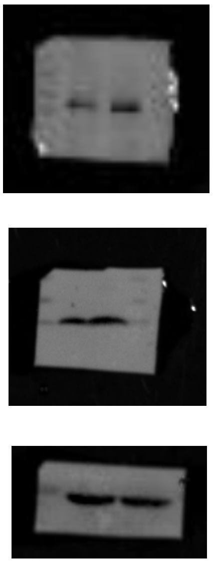

Fig. 7 | RP11-54O7.17 promotes autophagy-lysosome degradation of S100A4 through the binding of repeat fragment P to S100A4.

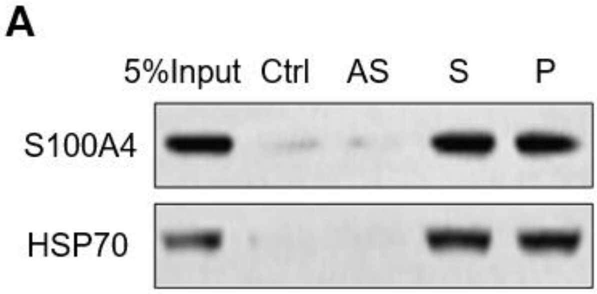

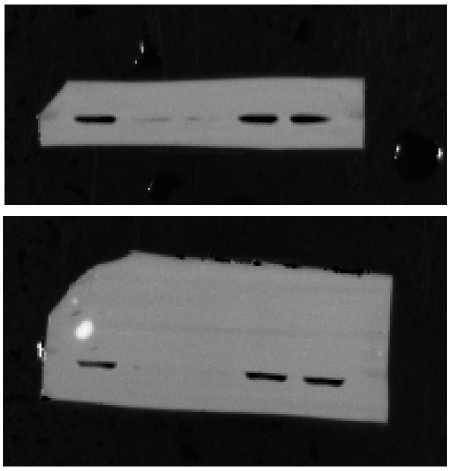

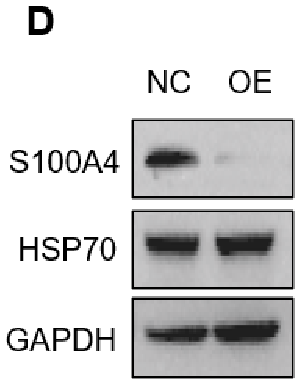

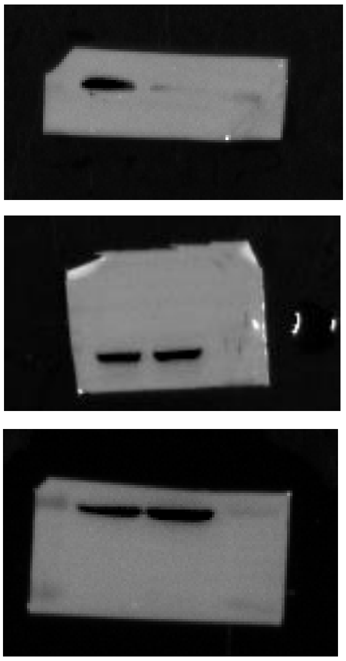

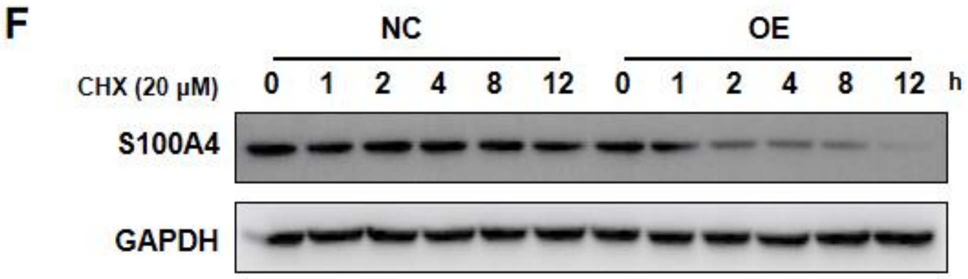

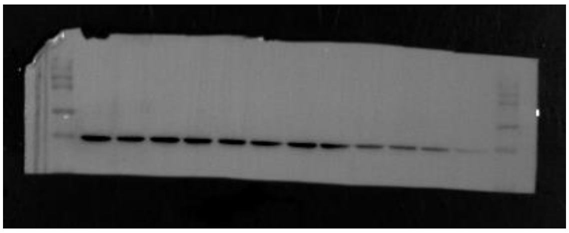

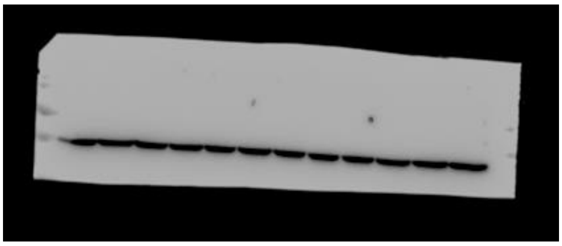

Fig. 7 | RP11-54O7.17 promotes autophagy-lysosome degradation of S100A4 through the binding of repeat fragment P to S100A4.

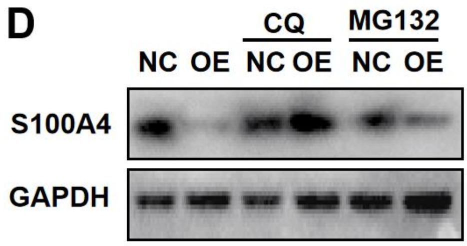

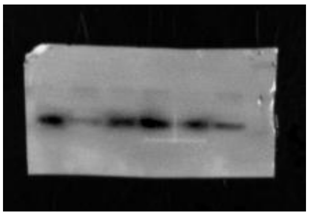

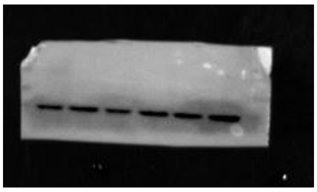

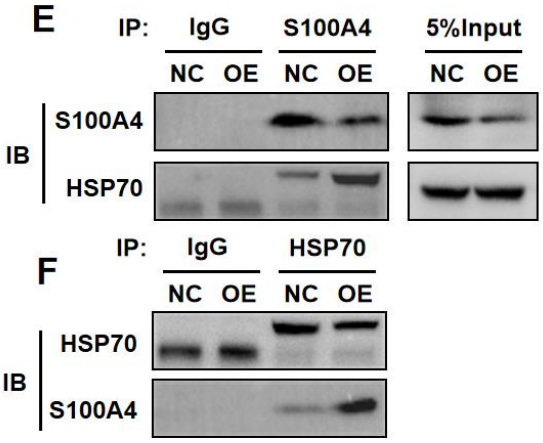

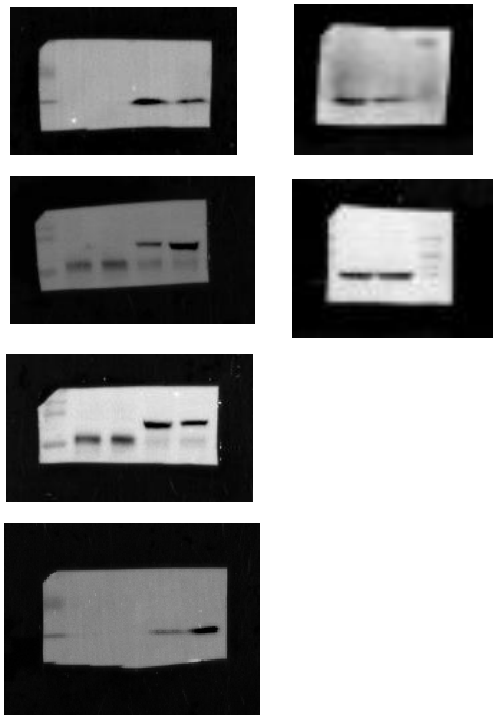

Fig. 8 | RP11-54O7.17 liposome suppresses TNBC growth in vivo.

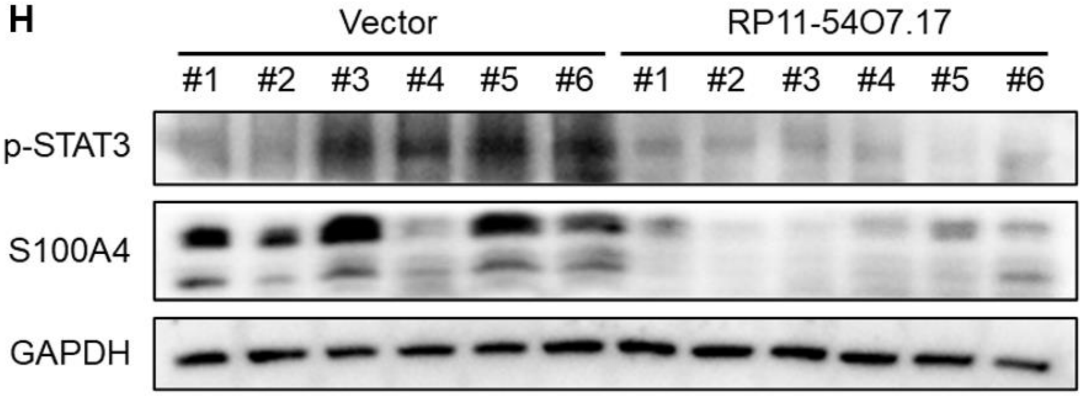

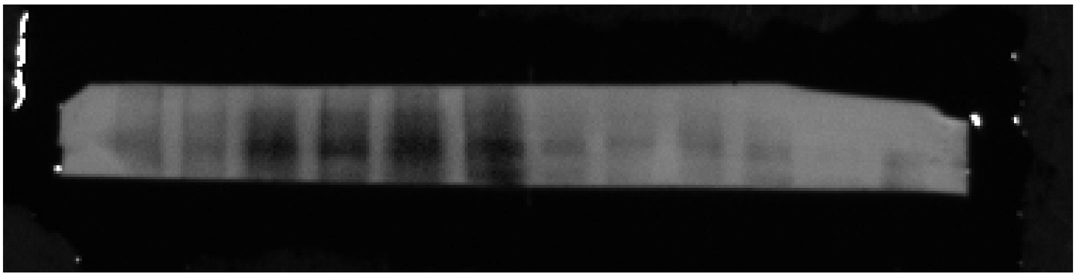

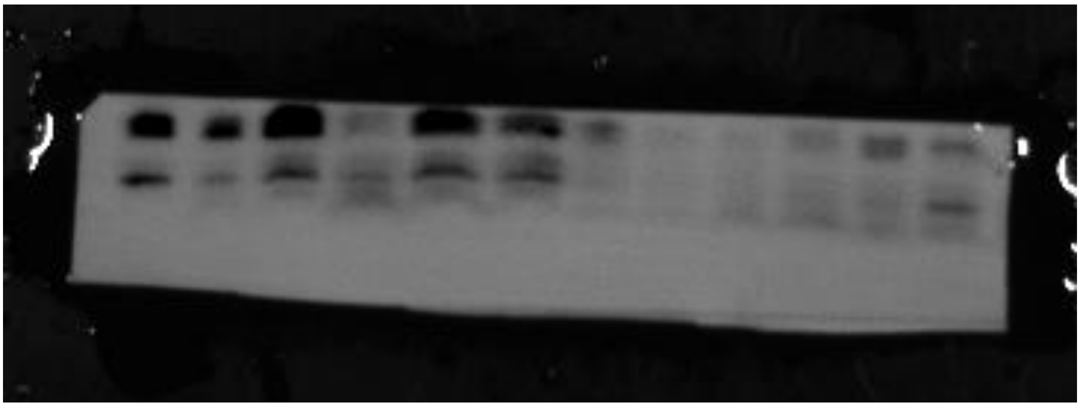

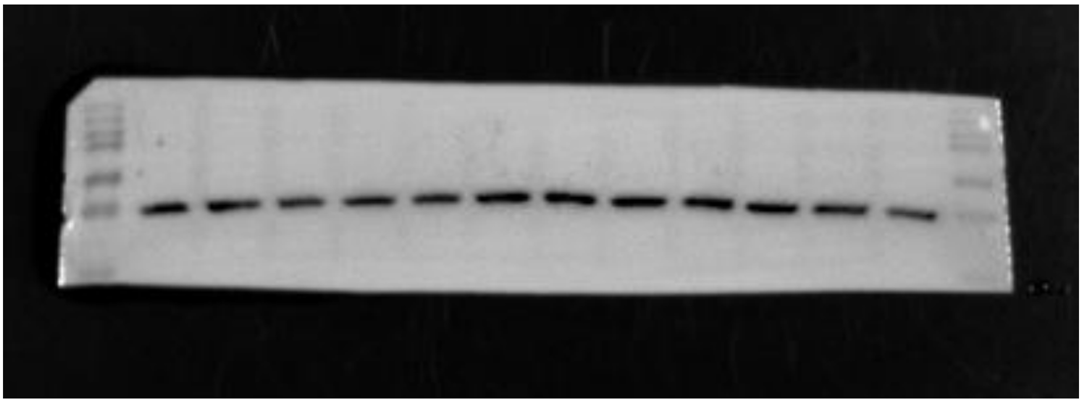

I

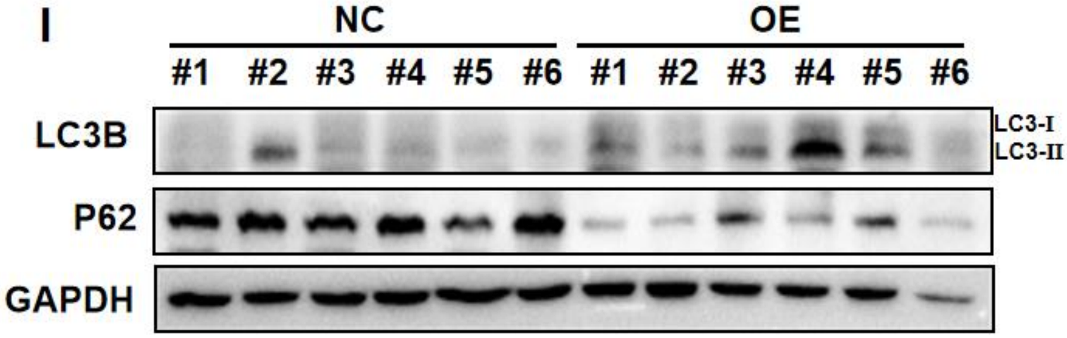

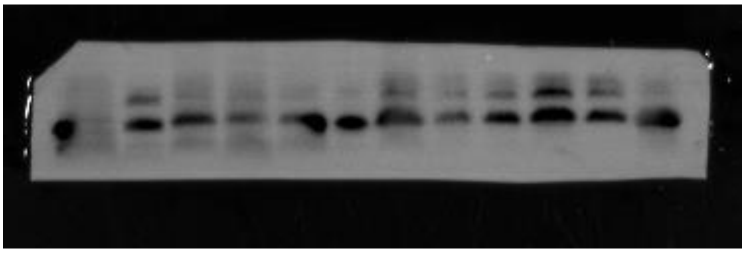

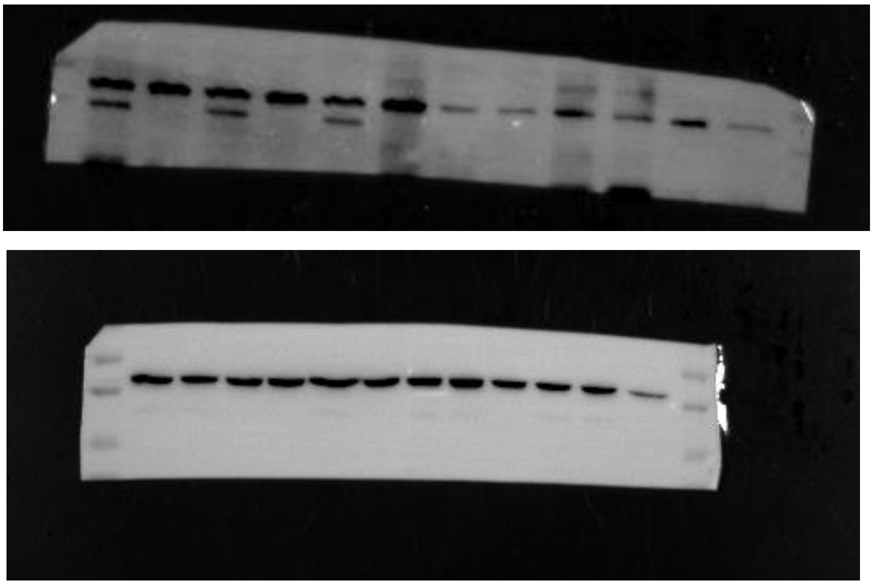

Supplementary Fig. 5 | Binding fragment of RP11-54O7.17 to S100A4 detected by RIP assay.

A

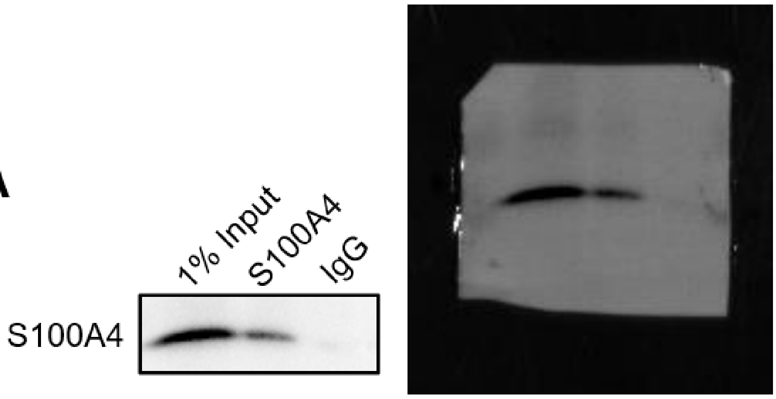

C

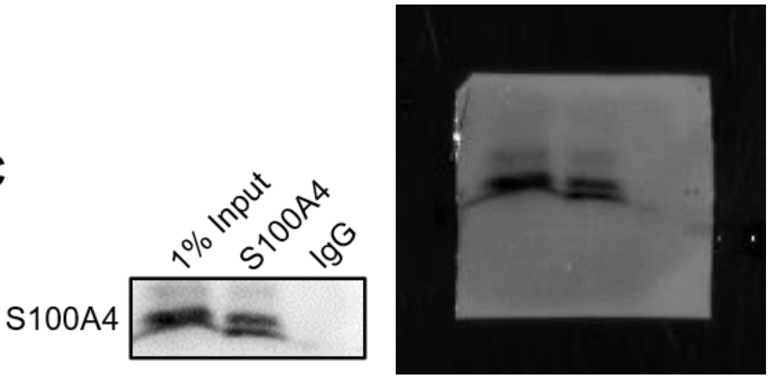

Supplement: Supplementary file 8 — Full and uncropped western blots [file 41419_2025_8072_MOESM8_ESM.pdf]
